# Inferring modulators of genetic interactions with epistatic nested effects models

**DOI:** 10.1371/journal.pcbi.1005496

**Published:** 2017-04-13

**Authors:** Martin Pirkl, Madeline Diekmann, Marlies van der Wees, Niko Beerenwinkel, Holger Fröhlich, Florian Markowetz

**Affiliations:** 1 ETH Zurich, Department of Biosystems Science and Engineering, Basel, Switzerland; 2 SIB Swiss Institute of Bioinformatics, Lausanne, Switzerland; 3 University of Amsterdam, Amsterdam, The Netherlands; 4 Bonn-Aachen International Center for IT (B-IT), University of Bonn, Bonn, Germany; 5 UCB Biosciences GmbH, Monheim, Germany; 6 University of Cambridge, Cancer Research UK Cambridge Institute, Cambridge, United Kingdom; University of Toronto, CANADA

## Abstract

Maps of genetic interactions can dissect functional redundancies in cellular networks. Gene expression profiles as high-dimensional molecular readouts of combinatorial perturbations provide a detailed view of genetic interactions, but can be hard to interpret if different gene sets respond in different ways (called *mixed epistasis*). Here we test the hypothesis that mixed epistasis between a gene pair can be explained by the action of a third gene that modulates the interaction. We have extended the framework of Nested Effects Models (NEMs), a type of graphical model specifically tailored to analyze high-dimensional gene perturbation data, to incorporate logical functions that describe interactions between regulators on downstream genes and proteins. We benchmark our approach in the controlled setting of a simulation study and show high accuracy in inferring the correct model. In an application to data from deletion mutants of kinases and phosphatases in *S. cerevisiae* we show that epistatic NEMs can point to modulators of genetic interactions. Our approach is implemented in the R-package ‘epiNEM’ available from https://github.com/cbg-ethz/epiNEM and https://bioconductor.org/packages/epiNEM/.

## Author summary

Genes do not act in isolation, but rather in tight interaction networks. Maps of genetic interactions between pairs of genes are a powerful way to dissect these relationships. Genetic interactions are mostly defined by quantifying individual phenotypes like growth or survival. However, when high-dimensional phenotypes are observed, genetic interactions can become very hard to interpret. Here we test the hypothesis that complex relationships between a gene pair can be explained by the action of a third gene that modulates the interaction. Our approach to test this hypothesis builds on Nested Effects Models (NEMs), a probabilistic model tailored to inferring networks from gene perturbation data. We have extended NEMs with logical functions to model gene interactions and show in simulations and case studies that our approach can successfully infer modulators of genetic interactions and thus lead to a better understanding of an important feature of cellular organisation.

## Introduction

More than 80% of genes in the yeast *Saccharomyces cerevisiae* are non-essential and the organism can survive their loss [1]. This observation points to the large number of functional redundancies built into the molecular networks of the cell. Maps of genetic interactions (also called epistasis) between pairs of genes are a powerful way to dissect these functional redundancies [2]. Generally, a genetic interaction is defined by the difference between the phenotype of a double-perturbation and the combined phenotypes of two single-gene perturbations [3, 4]. Costanzo *et al* [5, 6] comprehensively mapped the yeast genetic interaction network and the profiles they measured can be clearly associated to cellular functions [7].

While most genetic interaction maps use survival [8] or growth [2] as phenotypes, a more refined view of perturbation effects can be achieved by using high-dimensional molecular readouts like global gene expression [9]. A prominent example of this approach is the study by van Wageningen *et al* [10], who analyzed gene expression profiles of (combinations of) 150 deletion mutants of protein kinases and phosphatases in *S. cerevisiae*. They called the most common genetic interaction they found *mixed epistasis*, because different gene sets responded in different epistatic ways. Similar findings were later made in a larger follow-up study by Sameith *et al* [11]. Genetic interactions can be hard to understand mechanistically [12] and the complexity of mixed epistatic relationships makes their explanation particularly difficult.

### Definition of mixed epistasis

For two fixed knock-out mutations, we denote by 00 the wild type, by 10 and 01 the two single mutants, and by 11 the double mutant. Their effect on the expression of a given gene *i* is denoted by *E*_*i*,00_, *E*_*i*,01_, *E*_*i*,10_, and *E*_*i*,11_. Gene expression is reported as the log-fold change relative to the wild type 00, hence *E*_*i*,00_ = 0. Epistasis between the two mutations is defined as

$$\varepsilon_{i}=E_{i,00}+E_{i,11}-E_{i,01}-E_{i,10}=E_{i,11}-E_{i,01}-E_{i,10}.$$

Van Wageningen et al. consider this quantity over all effect genes *i* and define different types of epistasis for the multivariate gene expression phenotype (*E*_1_, …, *E*_*m*_).

Complete redundancy is the situation in which, for most genes *i*, *E*_*i*,01_ = *E*_*i*,10_ = 0 and hence *ε*_*i*_ = *E*_*i*,11_. Depending on the sign of *E*_*i*,11_, epistasis may be positive or negative for each individual gene *i*.

**Fig 1:**
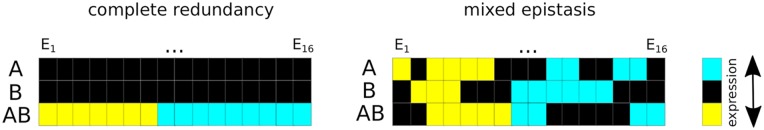
Schematic representation of different buffering relationships. Left: Complete redundancy is explained by an effect only being visible when both genes A and B are knocked out simultaneously. Right: Mixed epistasis is characterized by a mixed behaviour of two genes. Their interaction differs for different gene sets.

Mixed epistasis is defined by *E*_*i*,01_, *E*_*i*,10_ ≠ *E*_*i*,11_ for some genes and some of those not following redundancy. It is mixed in the sense that the single mutants can have any effect, positive or negative, in any combination (see Fig 1).

### Dissecting mixed epistasis

In this paper we test the hypothesis that mixed epistasis between a gene pair can be explained by the action of a third gene that mediates between the functional interaction and the transcriptional readout. To test this hypothesis, we extend the framework of Nested Effects Models (NEMs), which has been specifically tailored to analyze high-dimensional gene perturbation data [13]. The extended framework, called *Epistatic NEMs* (for short epiNEMs), incorporates logical functions that describe interactions between regulators. Our method is general and can be applied to all datasets that measure multi-parametric phenotypes for combinatorial perturbations. We benchmark the accuracy of epiNEMs in the controlled setting of a simulation study. In an application to the data of van Wageningen *et al* [10] and Sameith *et al* [11] we show that epiNEMs can point to mediators of genetic interactions.

### Previous approaches

There exist many different pathway reconstruction methods [14, 15]. Biological databases like BioGrid [16] construct their interaction networks by directly linking genes or proteins with known regulatory relationships, e.g. kinases and their substrates. Data-driven statistical measures like correlation [17] or mutual information [18] can be used to define edges between pairs of genes. Other probabilistic approaches for network inference are based on candidate graphs being evaluated according to the underlying data [14, 19]. Main representatives of this group are Bayesian and Boolean networks.

Boolean networks have a long tradition in biology [20] and were used to model signaling pathways [21] and reconstruct them from perturbations [22]. They model regulatory networks by allowing the nodes/genes to take on one out of two possible values (yes/no, on/off, expressed/not expressed). The choice of value depends on the states of the previous nodes/genes in the network. Boolean variables are dependent on conditional or logical statements and might change according to their input. Those statements are represented by a Boolean function that takes several Boolean variables as input, connects them with logical operators and results in one Boolean output value. In the context of mixed epistasis, van Wageningen *et al.* [10] used Boolean modeling in order to evaluate all possible combinations of connections between two nodes. This approach is fixed on two regulators with two corresponding gene sets and does not aim at network structure learning.

Bayesian networks have been used on multi-parametric readouts of gene perturbations [23, 24] and are flexible enough to capture complex interactions between regulators [25]. However, they require that most perturbation effects are measured directly at other pathway members, while in our setting the transcriptional effects are all measured downstream of the pathway of interest.

**Fig 2:**
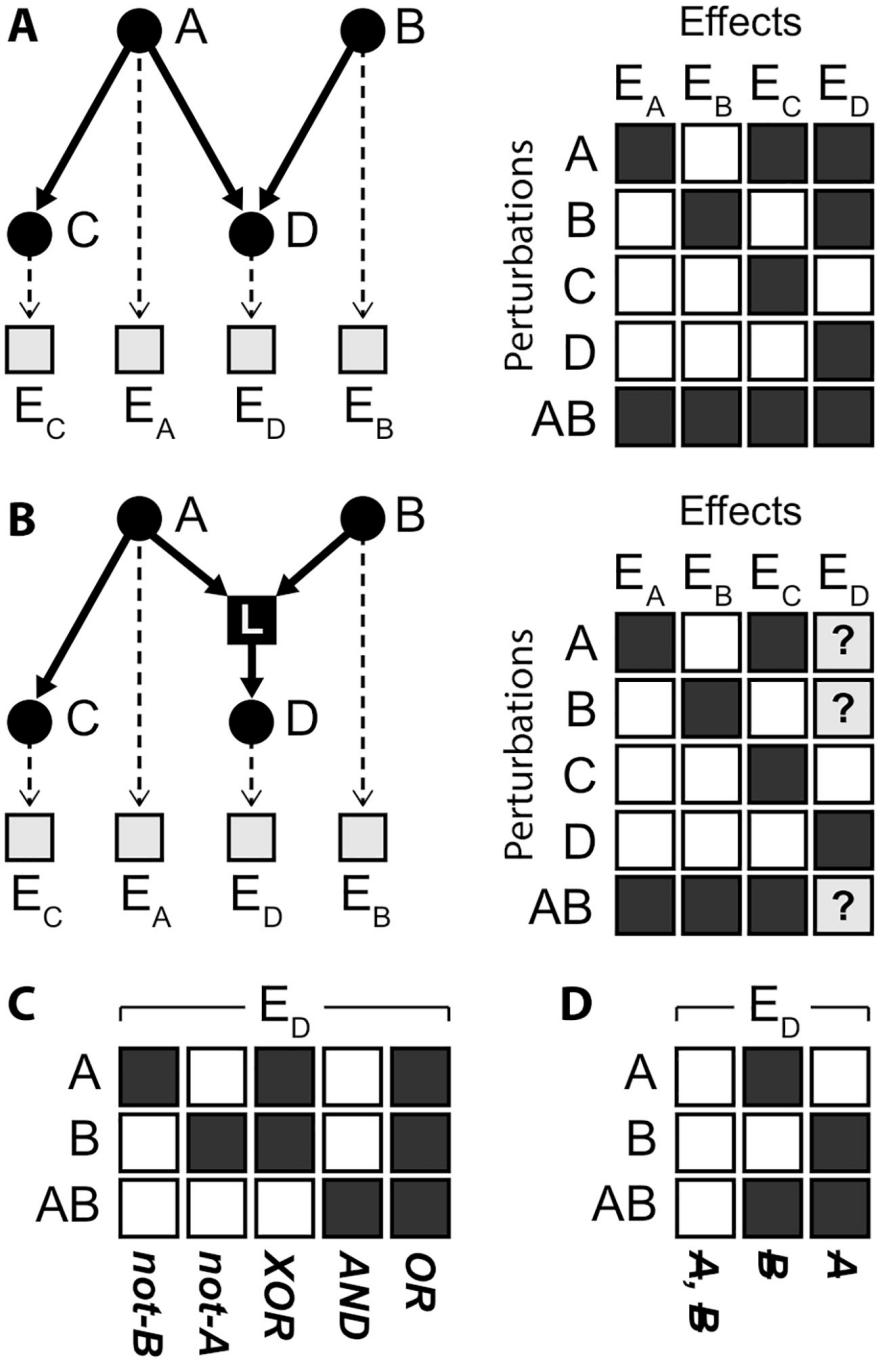
epiNEMs versus NEMs. **(A)** Nested Effect Models model how perturbations on signaling genes/proteins (*A*, *B*, *C*, *D*) affect downstream sets of effect reporters (*E*_*A*_, *E*_*B*_, *E*_*C*_, *E*_*D*_). Effects of perturbing *D* (=*E*_*D*_) are nested in the effects of perturbing *A* (= {*E*_*A*_, *E*_*C*_, *E*_*D*_}) and *B* (={*E*_*B*_, *E*_*D*_}). The matrices show the expected behaviour under the model. In real data, each gene in a set of effect reporters *E*. can be independently influenced by noise. **(B)** epiNEMs introduce logical functions for every node that has two parents (in this case *D*). The choice of logical function determines the effects observed in a combinatorial perturbation. The only difference to the NEM without logical functions is the expected perturbation effect on *E*_*D*_ if *A* or *B* are perturbed individually or in combination (indicated by question marks). **(C)** Five of the 2^3^ = 8 possible logical functions are AND, OR, XOR, not-A and not-B. The NEM in (A) is the special case of epiNEM with an OR logic. **(D)** The three other logical functions can be expressed by simpler graph structures, which remove an edge from A, or B or both.

This limitation motivated the development of Nested Effects Models (NEMs) to indirectly reconstruct signaling networks from observations of downstream genes whose expression levels are affected by perturbations of signaling proteins [26]. The name “Nested Effects Models” derives from the fact that NEMs infer directed relations between signaling proteins by the nested structure of subset relations between their perturbation effects (See Fig 2A). Since their introduction NEMs have been applied and extended in several case studies [27–33]. NEMs have also been extended to model pathway dynamics and re-wiring [34–37] as well as unobserved pathway activation [38] and confounders [39].

The key contribution in this paper is to extend NEMs by introducing logical functions modeling the effects of combinatorial perturbations. The fact that NEMs can easily be extended in this way shows their advantage over subset-based methods that are only defined on pairs of variables [40, 41]. The idea of incorporating logical functions was already introduced in Boolean NEMs (B-NEMs) [42] and is also widely used outside the NEM literature [43]. Our approach differs, however, in several important aspects. B-NEMs aim at learning large signaling pathways and achieve this by incorporating prior knowledge, which excludes full network reconstruction. B-NEMs are generalized to model any Boolean function with an arbitrary number of parents. Thus, without prior knowledge, B-NEMs have to tackle a large search space even for a relatively small number of signaling genes. This can impede the inference and the identifiability of the underlying network, which is modeled as a hyper-graph [42]. epiNEMs on the other hand are a straightforward extension of NEMs and model the pathway as a normal graph. If a signaling gene has two parents the incoming edges are annotated with one of five different logical functions. This aspect makes epiNEMs much more practical for handling the special case of epistasis, especially for large knock-out screens, where we test a multitude of single knock-outs (modulators) for several double knock-outs.

## Model

EpiNEMs consist of three elements (Fig 2B): First, a directed graph *G* between signaling genes *S*_*i*_. Second, a directed graph Θ linking each observed effect *E*_*i*_ to exactly one of the signaling genes *S*_*i*_. Combining these two graphs results in the NEM model. Third, in our epiNEM approach we add logical functions, one for each signaling gene *S*_*i*_ that has two or more parents in *G*.

### Logic gates

In total there are five logic gates that represent different biological relationships (see Fig 2C). The AND gate accounts for functional overlap of two genes. The pathway can compensate the loss or knock-down of one gene and only if both parents are off at the same time, the signal flow will be cut off. NOT-A and NOT-B stand for masking or inhibiting effects. The XOR gate can be interpreted as both parent genes preventing each other from acting on the third gene. The OR gate is identical to how two interactors are treated in the classic NEM approach: no interaction. All other theoretically possible logical combinations can be expressed in a simpler graph structure and are therefore disregarded (see Fig 2D).

### Boolean networks

Adding logics extends the *S*-gene graph into a Boolean network. In general, Boolean networks are dynamical systems, which can exhibit different attractors and steady states [44]. Our implementation covers this general case and uses the R package ‘BoolNet’ [44] to compute attractors and steady states for each single and each double knock-out in a synchronous manner. However, the assumptions we can make for the specific application of identifying modulators of genetic interactions guarantee a single steady state per network. First of all, we assume that effects on signaling genes due to direct or upstream perturbations are irreversible, which prevents feedback loops. Secondly, in our screens for modulators we only evaluate acyclic networks of three genes.

Thus, each set of perturbations corresponds to a unique pattern of activation states of pathway genes and we can summarize the expected effects on pathway genes in a row-vector *ϕ*. Concatenation of these vectors for all perturbations yields a design matrix Φ, in which the rows indicate expected effects for each perturbation.

### Inference in epiNEMs

Given the states of all signaling genes *S*_*i*_, we calculate the likelihood of each model hypothesis in the same way as in standard NEMs [26]. Let us first assume that the complete model, i.e. the signaling graph Φ and the effect attachments Θ = {*θ*_1_, …, *θ*_*m*_}, is given. With these parameters, the expected effects can be compared to the observed effects to obtain the likelihood

$$P\left(D|\Phi ,\Theta \right)=\prod_{i=1}^{m}\prod_{k=1}^{l}P\left(e_{ik}|\Phi ,\theta_{i}\right),$$

where *m* denotes the number of effects and *l* stands for the number of replicate experiments. For the effects, we have *e*_*ik*_ = 1 if we observe an effect and *e*_*ik*_ = 0 if we do not observe any effect. Experimental data, however, will always be noisy and therefore the probability *P*(*e*_*ik*_|Φ, *θ*_*i*_) will be dependent on the false positive rate *α* and false negative rate *β* of the experiment.

In almost all applications, however, it is not known which effect is directly linked to which signaling gene. Therefore, the marginal likelihood for each silencing scheme is computed by averaging over the effects attachments Θ. This is achieved by summing over all attachment probabilities:

$$P\left(D|\Phi \right)=\frac{1}{n^{m}}\prod_{i=1}^{m}\sum_{j=1}^{n}\prod_{k=1}^{l}P\left(e_{ik}|\Phi ,\theta_{i}=j\right),$$

where *n* denotes the number of signaling genes *S*_*i*_. The optimal pathway is the one resulting in the highest likelihood. For small networks like the ones we use here, exhaustive search over all network topologies is possible. For faster inference or feasibility for networks with more than five genes, a greedy hill climbing method is provided in the package ‘epiNEM’.

### Challenges in network inference

Interpretation of the network inferred from data is not always straightforward. As in the original NEM approach we have to consider the degree of identifiability of the network. Two networks belong to the same equivalence class if they have the same likelihood given the data. In the case of the original NEMs two networks are equivalent, if they have the same transitive closure. Due to our extension of the method, additional equivalences between network hypotheses occur in the case of epiNEMs.

Let Φ be a network with two parents regulating their child by one of epiNEMs‘ five logics or one of the three other types of relations (Fig 2C and 2D). If the parents are independent of each other, all eight networks result in different effect matrices and subsequently different data. However, if the parents are not independent, i.e., one parent is regulating the other parent, a knock-out of the upstream parent is equivalent to the double knock-out of both parents. Thus, networks which are only distinguishable by the effects of the single knock-out of the upstream parent become equivalent and produce the same data.

Another major challenge in pathway inference methods are hidden players [39]. In the case of NEM, if two parents have a hidden common child, the data shows all possible pairwise effects, i.e., effect reporters which react exclusively to one parent’s knock-out and effect reporters which react to both knock-outs. EpiNEMs are designed to use large knock-out screens to identify those hidden signaling genes as modulators of the signal and explanation of the corresponding data.

## Results and discussion

We validated and benchmarked epiNEMs in the controlled setting of a simulation study. In two case studies in *S. cerevisiae* we show that epiNEMs can identify potential modulators of genetic interactions.

### Benchmarking and validation of epiNEMs

In a simulation study, we compare epiNEM results to networks reconstructed by NEM without logics as well as B-NEM, ARACNE [18] (a method based on mutual information), and the PC algorithm [45] (a method based on partial correlation).

We generated data sets of 4-node networks with 100 effects, *E*_*i*_, being randomly attached to the 4 signaling genes, *S*_*i*_. In each network, two of the four signaling genes were randomly connected by one of the five possible logic gates. These networks were translated into adjacency matrices with knock-outs in the rows and observed signal disruptions in the columns. For every *E*_*i*_ we check the behavior upon perturbation from the adjacency matrix. We kept the false positive rate *α* at 0.1 and varied the false negative rates *β* over a wide range of values: *β* ∈ {0.01, 0.025, 0.05, 0.1, 0.2, 0.3, 0.4, 0.5}.

In total, we generated data from 100 random networks, for each false negative rate. We compared the five competing methods by running time and accuracy of the predicted edges. In the case of the PC algorithm and ARACNE, we did not consider the edge direction, because they only infer partially directed and undirected networks, respectively. For B-NEM and epiNEM, we additionally scored the accuracy of the inferred logical gates and their expected data generated by the inferred network, which is similar to the truth table of a Boolean network.

**Fig 3:**
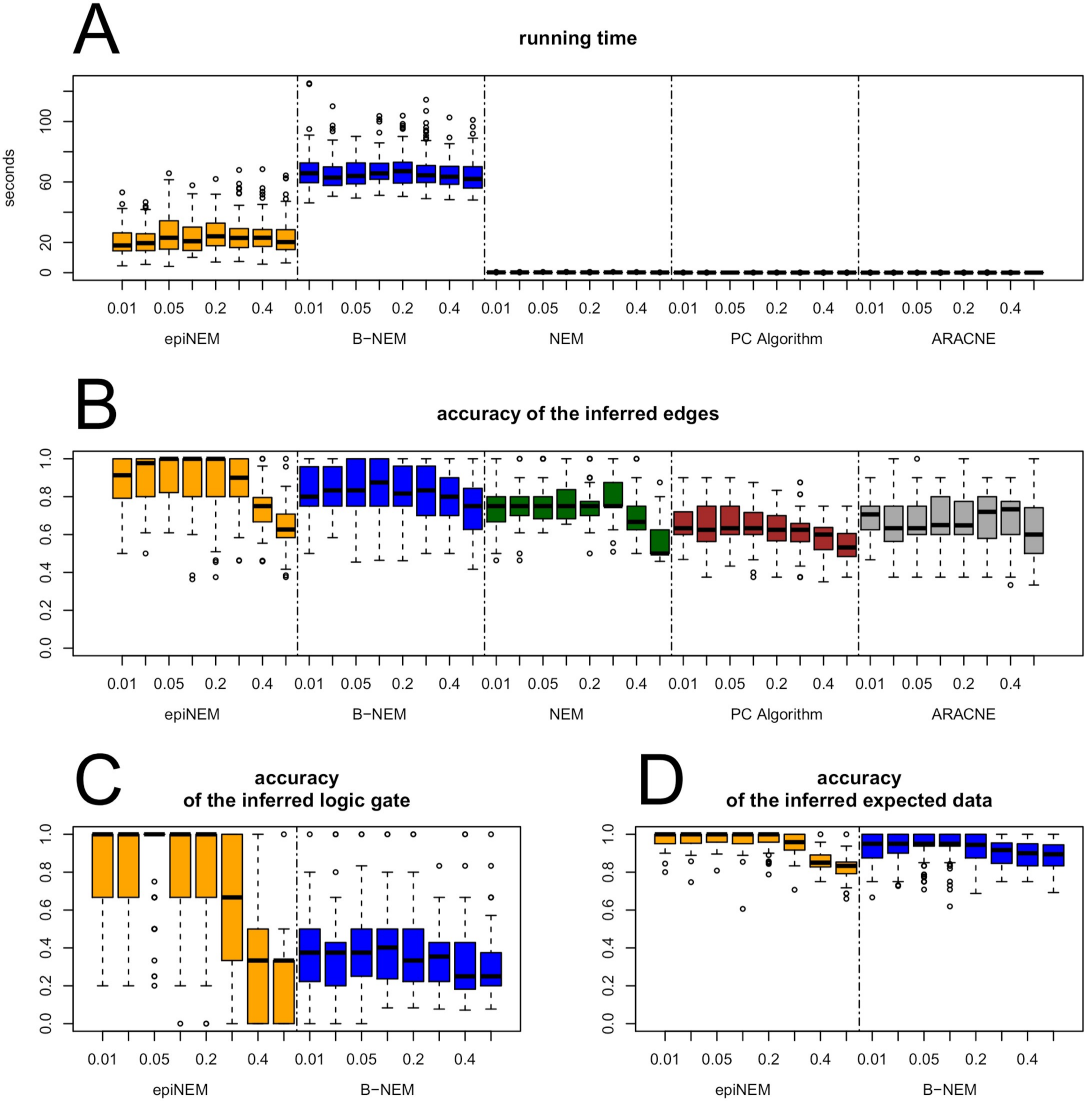
Result of 100 simulation runs on 4 node networks. (A) Time in seconds. (B) Accuracy of inferred edges. Accuracy of logic gates (C) and expected data (D), which is similar to the truth table. epiNEM is faster than B-NEM and slower than the other methods, while correctly identifying the logic gate for the median of all networks for up to 20% of false negative rate.

ARACNE, the PC algorithm, and NEMs are by far the fastest methods. However, they do not infer any logical gates, and the first two report no or only partial edge directions, respectively. B-NEM is almost a magnitude slower than epiNEM. Additionally, epiNEM achieves the highest accuracy for the inferred edges, closely followed by B-NEM and with some distance the other methods. Due to B-NEM’s larger search space, it cannot identify the correct epistatic signaling, even though the accuracy for the expected data is high. EpiNEM on the other hand, while only achieving little higher accuracy for the expected data, has median accuracy of 100% for the logic gates and false negative rates up to 20% (see Fig 3).

### EpiNEMs infer modulators of mixed epistasis

We applied epiNEMs to the studies of yeast knock-out screens of van Wageningen *et al.* [10] and Sameith *et al.* [11]. Both data sets consist of measurements of gene expression changes from double and single gene knock-out experiments in *S. cerevisiae*. Our goal is to identify signal modulators that help explaining the mixed epistasis patterns observed under single and double knock-outs of signaling genes.

Van Wageningen *et al.* identified three buffering relationships: quantitative redundancy, complete redundancy, and mixed epistasis [10]. The last case is the most prevalent and defined by two genes interacting in different epistatic ways for different downstream gene sets. Mixed epistasis suggests that genes may only partially overlap in function or be influenced by an additional regulatory module that controls different processes according to condition and environment.

#### Screening for modulators

Taking up the idea of such a modulator, we used epiNEMs to screen genetic interactions against every single mutant showing an effect compared to wild type. Each screen consisted of scoring many 3-node networks combining the two genes in the genetic interaction with a third gene from a set of potential modulators. To test the full range of possibilities, our search space contains models with and without logics. In order to be able to compare the marginal likelihood of the different models, a common set of $E_{i}$ for each genetic interaction was built as the union of effects over all single perturbations plus the effects of the respective double perturbation.

We used binarized data and thus only address complete redundancy and mixed epistasis. We do not restrict the search space to enforce epistasis, but allow every network hypothesis. However, for almost all significant modulators epiNEM infers logic gates. Only for some epiNEMs, we find “no epistasis”, defined as either some other type of regulatory network without logic gates or an unconnected network even though the three signaling genes share effect reporters. This is in contrast to “no information”, when the modulator does not share effect reporters.

#### Complete redundancy

**Fig 4:**
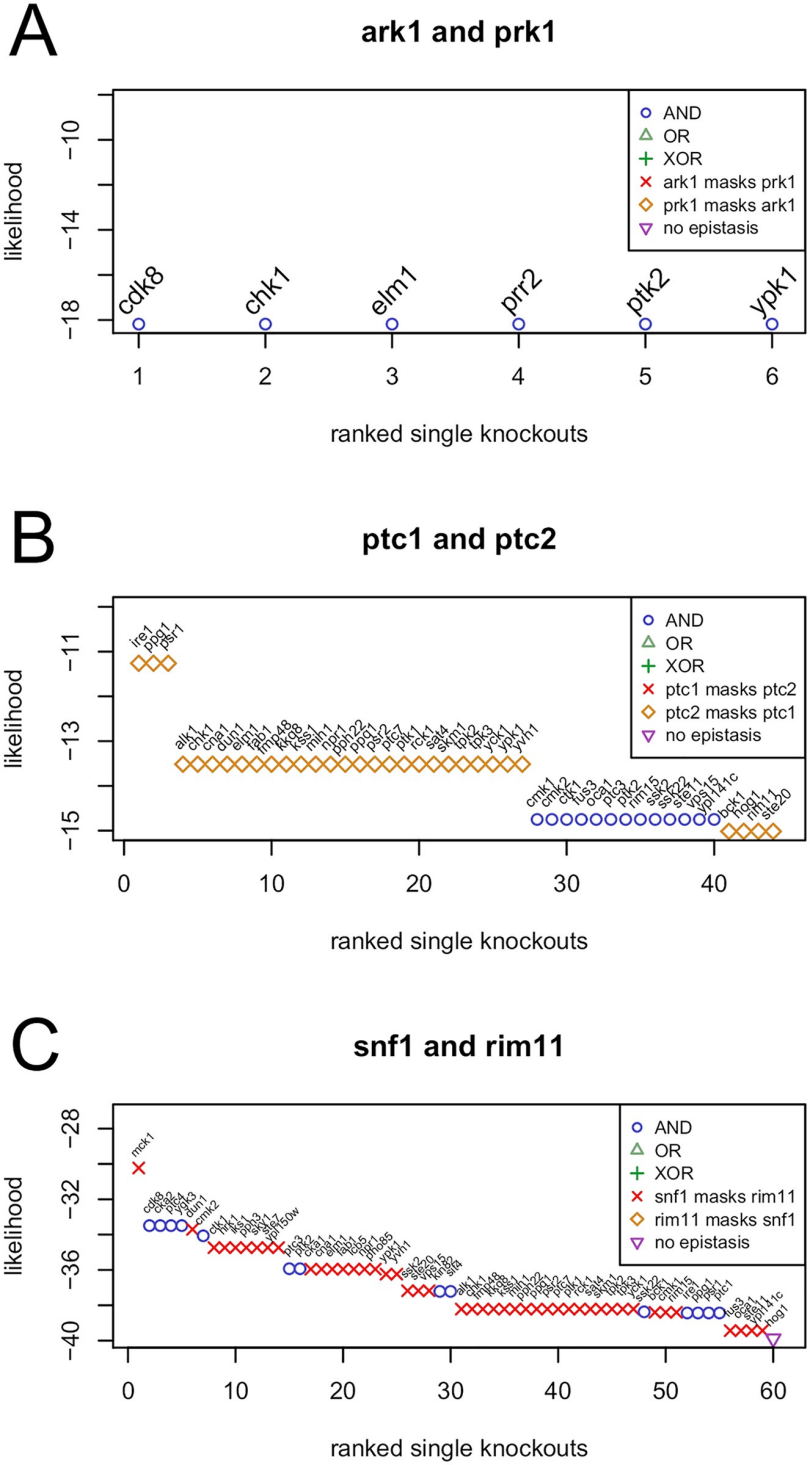
Identification of signal modulators. (A) The identified modulators for *ark1* and *prk1* confirm the complete redundancy. (B) The identified modulators for *ptc1* and *ptc2* exhibit masking of *ptc1* by *ptc2* and some lower ranking modulators complete redundancy. (C) The modulators of the *snf1* and *rim11* knock-out signal are identified as complete redundancy and the masking of *rim11* by *snf1*.

There are three pairs of double knock-outs, which were previously identified to exhibit complete redundancy [10], namely *ark1* and *prk1*, *ptp2* and *ptp3*, as well as *hal5* and *sat4*. Our analyses agree with these previous results (Fig 4A and vignette of package ‘epiNEM’). For *ark1* and *prk1*, epiNEMs identify six modulators, all regulated by complete redundancy (*cdk8, chk1, elm1, prr2, ptk2* and *ypk1*).

#### Quantitative redundancy

Another two pairs, *pctc1* and *ptc2*, and *ptc1* and *pph3*, of double knock-outs were previously identified as showing quantitative redundancy. Because epiNEMs use binary data, they are not designed to infer quantitative relationships. However, among the quantitative redundant gene pairs epiNEM identified almost exclusively masking relationships for all high scoring modulators. Only in the case of *ptc1* and *ptc2* some lower scoring modulators show complete redundancy (Fig 4B).

#### Mixed epistasis

For the remaining 9 double mutants, mixed epistasis was found by van Wageningen *et. al.* EpiNEM mostly confirms those findings, i.e., for almost all double mutants we found multiple logical functions for multiple modulators. However, in most cases, the regulations are dominated by one or two different logical functions, e.g., complete redundancy mixed with a masking effect (Fig 4C). Additionally, for *hsl1* with *cla4*, and *slt2* with *ptp3*, epiNEMs do not infer mixed epistasis, but complete redundancy.

#### Growth based genetic interactions

Sameith *et al.* produced 72 double knock-outs, which is roughly five times more than in the previous case study. They selected these pairs as they were previously identified as growth based genetic interactions with similar DNA binding properties. We used epiNEMs to perform the same screening for modulators on these pairs as before.

Sameith *et al.* put their focus on one example of a pair of antagonists namely *gln3* (activator) and *gzf3* (repressor). They identify the *gln3* mutant as affecting growth due to the repressed expression of many downstream target genes. *gzf3* has no effect on growth and results in only a few expression changes. However, among these few genes, they find the increased expression of *gat1*, which is like *gln3* an activator of transcription. The double mutant of *gln3* and *gzf3* shows no growth defect, which suggests a masking of the positive effect of *gln3* by *gzf3*. Their explanation is a derepression of *gln3* targets by *gzf3*.

**Fig 5:**
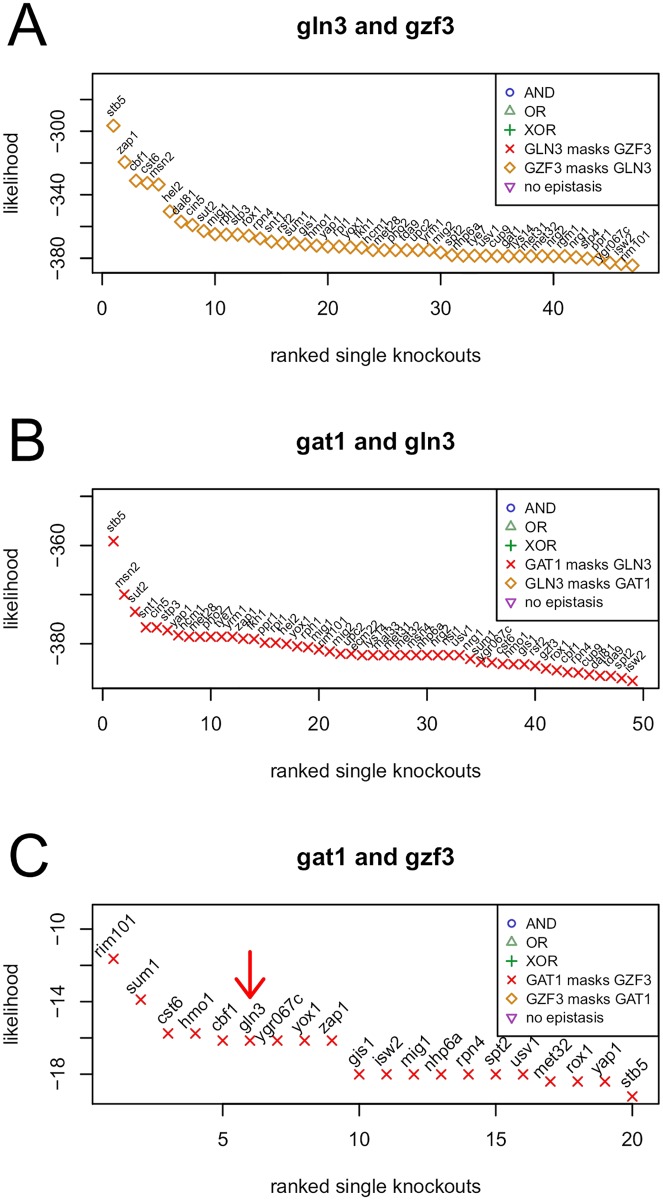
Interplay of *gln3, gzf3* and *gat1*. *Gzf3* masks the effect of *gln3* (A), which confirms the result of Sameith *et al*. *Gat1* masks both the effects of *gln3* and *gzf3* (B-C). Additionally, we identify *gln3* as a high scoring modulator of the signaling between *gzf3* and *gat1* (C, red arrow).

Fig 5A shows that epiNEMs identify over 40 modulators exclusively as “*gzf3* masks the effect of *gln3*”, which confirms previous results by Sameith *et al*. However, all modulators for the *gat1*-*gln3* double mutant are also identified as “*gat1* masks the effect of *gln3*” (Fig 5B), which indicates that *gat1* has a similar derepression effect on *gln3* target genes as *gzf3*.

For the *gat1*-*gzf3* double mutant, epiNEMs find less modulators (Fig 5C), which is in accordance with the few expression changes for the single *gzf3* knock-out discovered by Sameith *et al.* Interestingly, we again identify *gat1* as an inhibitor, but this time *gat1* is inhibiting *gzf3*. Furthermore we find *gln3* among the top modulators of the *gat1*-*gzf3* signal with the fourth highest likelihood (Fig 5C, dark red arrow). These results hint at *gat1* as having an important role not only as an activator of transcription, but also balancing gene expression changes between antagonists like *gln3* and *gzf3*.

#### Global distribution of logics

**Fig 6:**
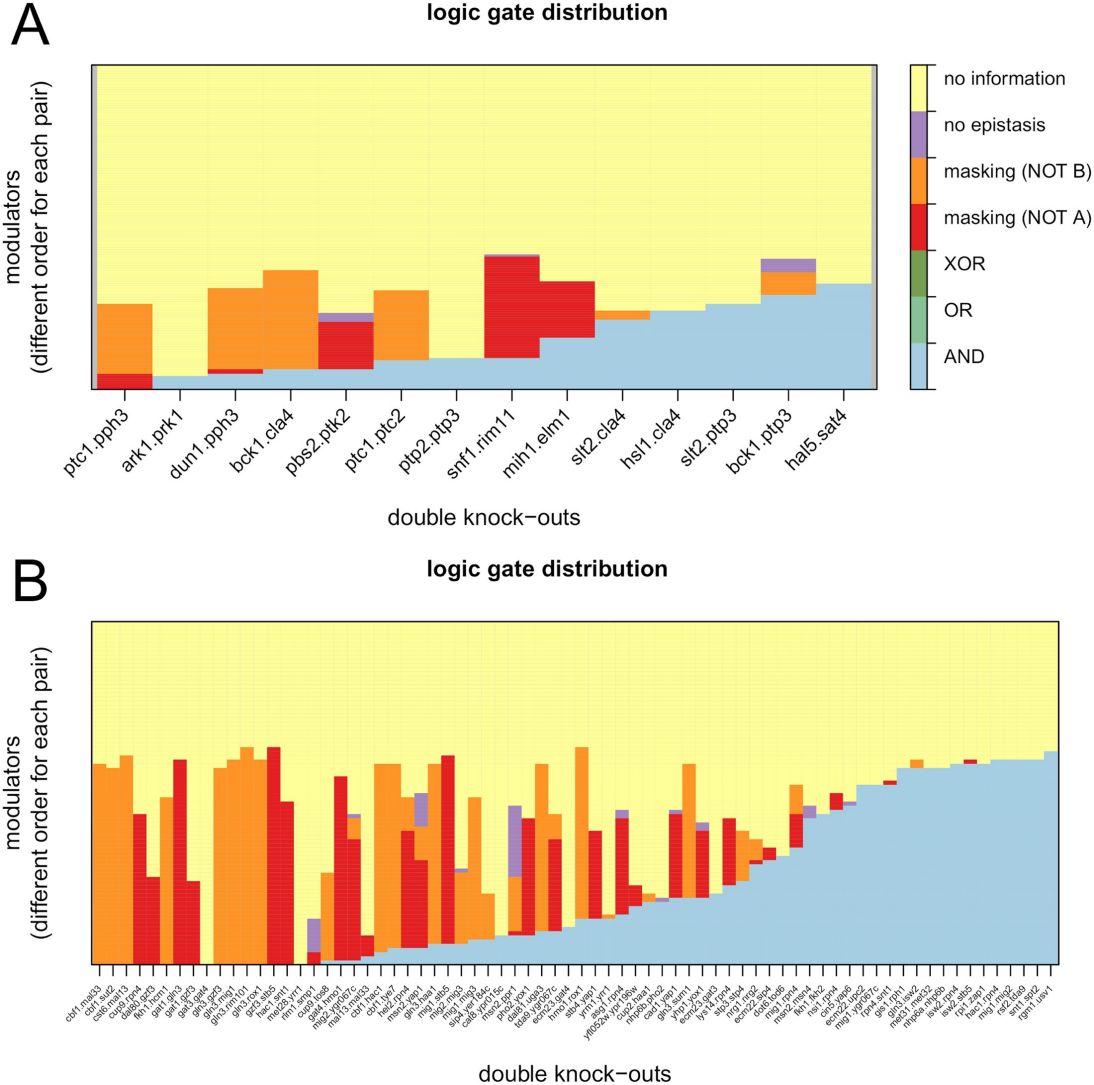
The distribution of the logic gates for each double knock-out of the data from van Wageningen *et al.* (A) and Sameith *et al.* (B). In both cases the AND logic (blue) is the most dominant. The absence of OR gates can be explained by the selection of regulators. Only a few modulators are identified as related to the regulators, but not via any logic (purple). False negatives in the data and equivalences can be responsible for the absence of XOR gates and the large amount of masking logics.

Fig 6 shows the distribution of the logic gates identified for each double knock-out. If the effect reporters reacting to a modulator do not react to the genes in the double knock-out, epiNEMs cannot infer any relationship and we list this as “no information” (Fig 6, yellow). OR and XOR gates are completely absent, which is something we expect for OR gates, because van Wageningen *et al.* and Sameith *et al.* selected double mutant pairs based on redundancy and mixed epistasis. A moderate to high false negative rate or equivalences we mentioned before can be responsible for XOR logics to be identified as masking effects. Several double knock-outs exclusively identify modulators for AND gates and several for a masking effect. The remainder identify mixed epistasis, often dominated by one masking logic.

Only a small fraction of modulators are not identified as modulating any epistatic effect (Fig 6, purple).

#### EpiNEMs identify modulators with significant interactions

**Fig 7:**
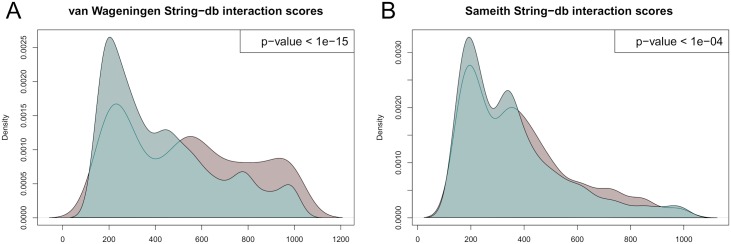
String-db interaction score distributions. The distributions for the string-db interaction scores for the top 30 modulators with their respective regulators (red) and the distributions for the interaction scores of all possible modulators and regulators in the data (blue) for the Van Wageningen *et al.* (A) and the Sameith *et al.* (B) data sets. The Mann-Whitney test with alternative “greater” produces p-values which indicate that the modulators identified by epiNEMs have higher interaction scores with their regulators than explained by random drawing.

To further validate that the modulators we inferred are biologically meaningful, we made use of the STRING [46] database of functional protein interactions. The interaction score between 0 (lowest) and 1000 (highest) comprises information from literature mining, experimental validation, cooccurence, genomic neighborhood, curated databases, coexpression and gene fusion. Thus, it is a general measure of interaction(s) and provides additional evidence for novel findings. Fig 7(A) and 7(B) shows the distribution of the string-db interaction scores between the top 30 modulators with their respective regulators for the van Wageningen *et al.* (Fig 7A, red) and the Sameith *et al.* (Fig 7B, red) data sets. The Mann-Whitney test shows that these distributions differ significantly with alternative “greater” from their respective interaction score distributions for all possible modulators and regulators (blue) in the data set. Thus, our identified modulators achieve higher interaction scores with their regulators than explained by randomly drawing two genes from the combined set of modulators and regulators.

We did the same analysis using a graph based GO similarity score (Wang et al., 2007 [56]) implemented in the R-package GOSemSim (Yu *et al.*, 2010 [57]) and achieved similar results (Fig. L and M in S1 Text).

Additionally, we performed KEGG pathway enrichment analysis for the set of significant modulators of each double knock-out pair as well as the effect reporters connected to each significant modulator. The modulators are highly enriched in common pathways like meiosis, cell cycle and MAPK signaling for both data sets (Fig. N and O in S1 Text). However, epiNEM identifies modulators for some double knock-outs of the van Wageningen *et al.* data set, which are enriched in a more unique set of pathways like glycerophospholipid metabolism. The enrichment analysis for the effect reporters shows a more uniform enrichment by a large number of pathways (Fig. P and Q in S1 Text). However, this can be explained by the fact, that the modulators themselves are involved in the pathways, while the effect reporters are responsible for secondary or even tertiary effects and thusly reach a much larger set of different pathways.

### Conclusion

In this paper, we have developed a method to address a central question of molecular cell biology: how to characterise the mechanisms underlying the functional redundancies visible in genetic interactions. We hypothesized that mixed epistatic effects found in high-dimensional readouts can be explained by the action of a third gene that mediates between the genetic interaction and the transcriptional response. To explore this hypothesis we extended Nested Effects Models, an established methodology to infer signaling pathways, with logical functions. The resulting method, called epiNEMs, is a general approach to infer pathways including combinatorial regulation from perturbation effects. In particular, it allowed us to screen for modulators of genetic interactions in *S. cerevisiae*. We were able to identify such modulators and to computationally reproduce the experimental results from van Wageningen *et al.* in most cases. In a second data set from Sameith *et al.* consisting of roughly five times more double knock-outs, we calculated the global distribution of epistatic signaling logics of all modulators. Most of them are identified as masking or complete redundancy. Additionally, we thoroughly investigated a previously by Sameith *et al.*, 2015 identified triplet of growth inducing and repressing factors *gln3, gat1* and *gzf3* and found evidence for a more complex signaling network. Furthermore, we globally visualized our findings by gene ontology enrichment analysis (KEGG pathways) to support the validity of epiNEMs.

Our approach has several limitations. First, extending NEMs with logics increases the size of the model space and makes exhaustive enumeration unfeasible. Second, we only consider logics between pairs of regulators, which helps to limit model space and is very well suited for our application to genetic interactions, but might be an oversimplification in other applications. In the future, the model could therefore be improved by allowing logic gates for more than two parents. This will result in more complex logics but will also allow for capturing more interactions. Also, until now it is only possible to distinguish between complete redundancy and mixed epistasis, while quantitative redundancy cannot be captured. To improve this situation, we plan to extend the model to use quantitative effects rather than binary data.

In summary, we presented a general framework to understand mediators of complex phenotypes of genetic interactions. Our case studies on transcriptional phenotypes in yeast showed very promising results and there are potentially many other applications in other organisms using either combinatorial RNAi [47] or pooled CRISPR screens [48] together with multi-parametric phenotyping [49] or single-cell RNA-seq [50–53].

## Materials and methods

### Implementation

All our analyses were done in the statistical computing environment R [54]. Our approach is implemented in the R-package ‘epiNEM’ available from https://github.com/cbg-ethz/epiNEM and https://bioconductor.org/packages/epiNEM/ [55].

### Data sets

We used 160 microarray gene expression profiles of single and double mutants from [10] available at ArrayExpress under the IDs E-TABM-907 (mutants) and E-TABM-773 (200 wild-type replicates). Additional we used 154 profiles from [11] with respective ID E-MTAB-1385. For our analyses, we directly downloaded the flat files from the following locations:

http://www.holstegelab.nl/publications/sv/signaling_redundancy/

http://www.holstegelab.nl/publications/GSTF_geneticinteractions/.

All analysis steps including data preprocessing are documented in the vignette of the R-package ‘epiNEM’.

## Supporting information

S1 Text The vignette of the epiNEM R-package containing supporting R code, figures and text. (PDF)
