# Supplementary material for: Inferring modulators of genetic interactions with epistatic nested effects models
Source: PLoS Comput Biol. 2017 Apr 13;13(4):e1005496. doi: 10.1371/journal.pcbi.1005496 (PMC5407847; doi:10.1371/journal.pcbi.1005496)
Supplement: S1 Text — (PDF) [file pcbi.1005496.s001.pdf]

Epistatic Nested Effects Models
Inferring mixed epistasis from indirect measurements of knock-out screens.

Martin Pirkl, Madeline Diekmann, Marlies van der Wees, Niko Beerenwinkel, Holger Fröhlich, Florian Markowetz

2017-04-06

Contents

0.1 Special Version: 1

1 Introduction 1

2 Loading epiNEM 2

3 Quick start 2

4 Simulations 4

5 Yeast knock-out screens 7

5.1 Identification of significant modulators for pairs of double knock-outs 7

5.2 Validation of identified interactions 8

5.3 Enrichment analysis 15

6 Creation of data objects 20

7 Session information 28

8 References 28

0.1 Special Version:

This version of the vignette is supplementary information for our publication in PloS Computational Biology. You can always find the newest version of the vignette as part of the epiNEM package on biocondoctor.org or github.

1 Introduction

The method in this package is an extension of the classic Nested Effects Models provided in the package *nem*. Nested Effects Models is a pathway reconstruction method, which takes into account effects of downstream genes. Those effects are observed for every knock-out of an upstream pathway gene, and the nested structure of observed effects can then be used to reconstruct the pathway structure. However, classic Nested Effects Models do not account for double knock-outs. In this package *epiNEM*, one additional layer of complexity is added. For every two genes, acting on one gene together, the relationship is evaluated and added to the model as a logical gate. Genetic relationships are represented by the logics OR (no relationship), AND (functional overlap), NOT (masking or inhibiting) and XOR (mutual prevention from acting on gene C). Please see the references for a more detailed description of NEMs and epiNEMs.

Simulation and application results are imported from pre-calculated data sets to shorten the runtime of this vignette.

2 Loading epiNEM

```
library(epiNEM)
```

3 Quick start

The data should be in the form of binary effects stemming from knock-out data including single and double perturbations with effect reporters, e.g. genes, as rows and perturbations as columns. A one in row i and column j denotes an effect of reporter i for perturbation j. These effects are usually derived from comparing control/wild-type experiments with respective perturbation experiments (e.g. differential expression with edgeR, Robinson *et al.*, 2010). The binary data is the main input for the epiNEM function, which contains the inference algorithm to estimate the underlying network structure of the data. For up to four signalling genes (=different perturbation targets) exhaustive search is available. For five or more a greedy search is implemented as an alternative.

```
data <- matrix(sample(c(0,1), 100*4, replace = TRUE), 100, 4)
colnames(data) <- c("A", "A.B", "B", "C")
rownames(data) <- paste("E", 1:100, sep = "_")
print(head(data))
##     A A.B B C
## E_1 1   1 0 0
## E_2 1   0 0 1
## E_3 1   0 1 1
## E_4 0   1 0 1
## E_5 0   0 0 0
## E_6 1   0 1 1
res <- epiNEM(data, method = "exhaustive")
```

```
plot(res)
```

The plot shows the inferred network with the signaling genes (bright red), the inferred logic (green) and attachment of the number of effect reporters (grey).

Alternatively the input can be a larger matrix with single and double knock-outs. EpiNEM can perform a systematic analysis to identify most likely modulators for a signaling gene pair of a double knock-out.

```
data <- matrix(sample(c(0,1), 100*9, replace = TRUE), 100, 9)
colnames(data) <- c("A.B", "A.C", "B.C", "A", "B", "C", "D", "E", "G")
rownames(data) <- paste("E", 1:100, sep = "_")
res <- epiScreen(data)
## [1] "A.B"
## [1] "A"
## [1] "B"
## [1] "C"
## [1] "D"
## [1] "E"
## [1] "G"
## [1] "A.C"
## [1] "A"
## [1] "B"
## [1] "C"
```

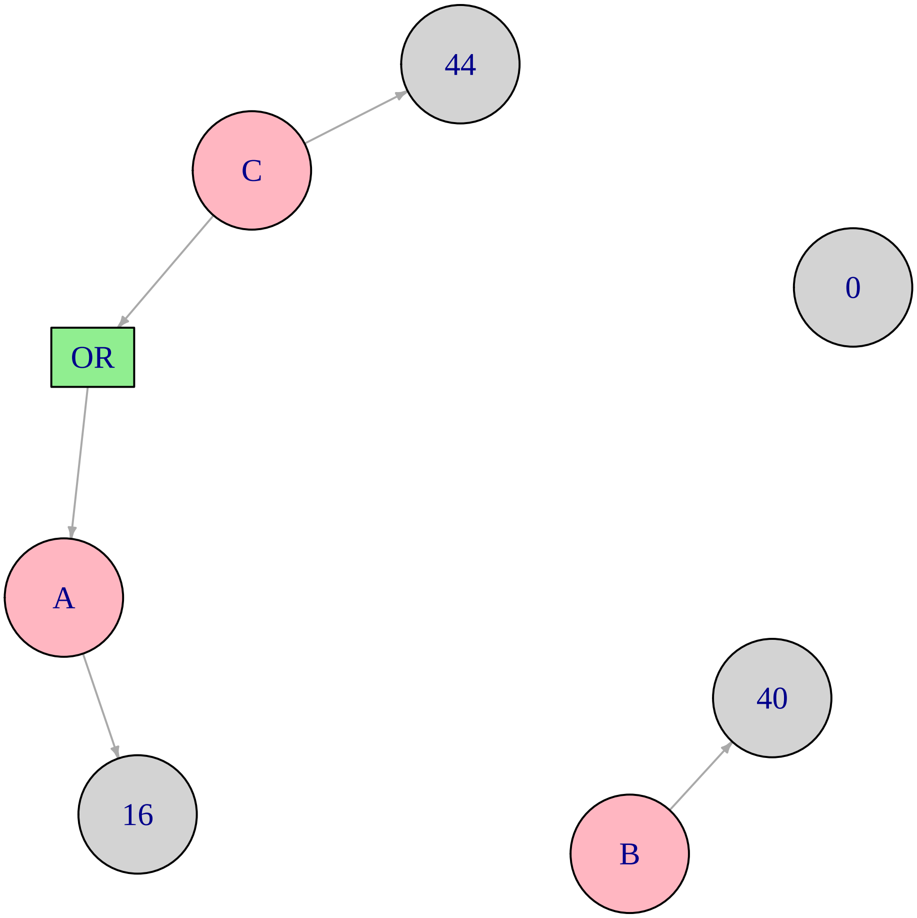

Figure A: Toy example result for a double mutant (A,B) and one modulator (C).

```
## [1] "D"
## [1] "E"
## [1] "G"
## [1] "B.C"
## [1] "A"
## [1] "B"
## [1] "C"
## [1] "D"
```

```
## [1] "E"
## [1] "G"
```

```
plot(res)
```

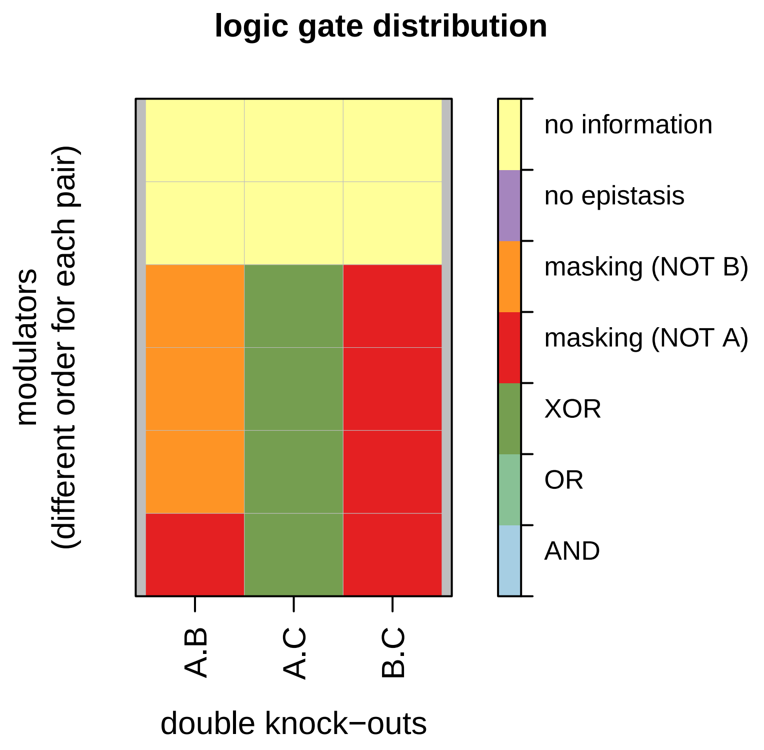

Figure B: Distribution of inferred logics for each double knock-out.

```
plot(res, global = FALSE, ind = 1)
```

If the "global" parameter is set to FALSE, detailed results are plotted for each or a specific ("ind" parameter) pair of a double knock-out.

The results (logics) of the knock-out screens have been annotated according to the following legend of effects (black), where the x-axis denotes the gene and the y-axis denotes the type of knock-out. E.g. if C is regulated via the OR gate, it is effected by by both single and the double knock-out.

```
epiAnno()
```

4 Simulations

We compare epiNEM to original NEMs (Markowetz *et al.*, 2005), Boolean Nested Effects Models (B-NEM, link, Pirkl *et al.*, 2016), Aracne (Margolin *et al.*, 2006) and the PC algorithm (Kalisch *et al.*, 2007) for 100 four node networks and respective data experiencing different levels of noise. The evaluation is done by accounting for the accuracy of discovered edges and in the case of B-NEM and epiNEM also the truth tables and logical gates.

We simulate code based on a ground truth and infer an optimal network with all aforementioned methods solely based on the data. The B-NEM package is necessary to run the full simulation: link. If the comparison to B-NEM is omitted, the B-NEM package is not necessary for epiNEM to function.

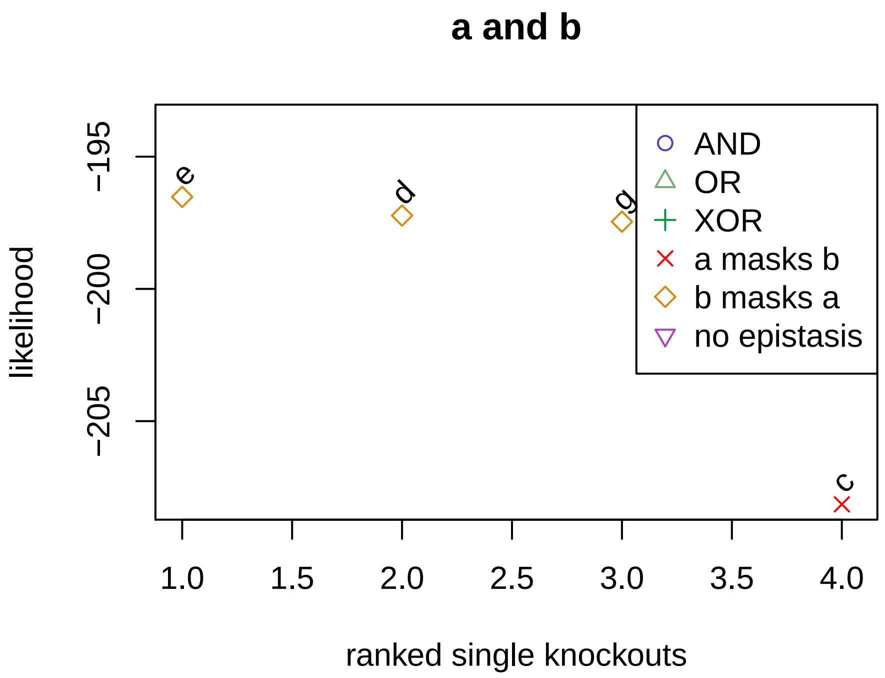

Figure C: Ranked modulators for one double knock-out.

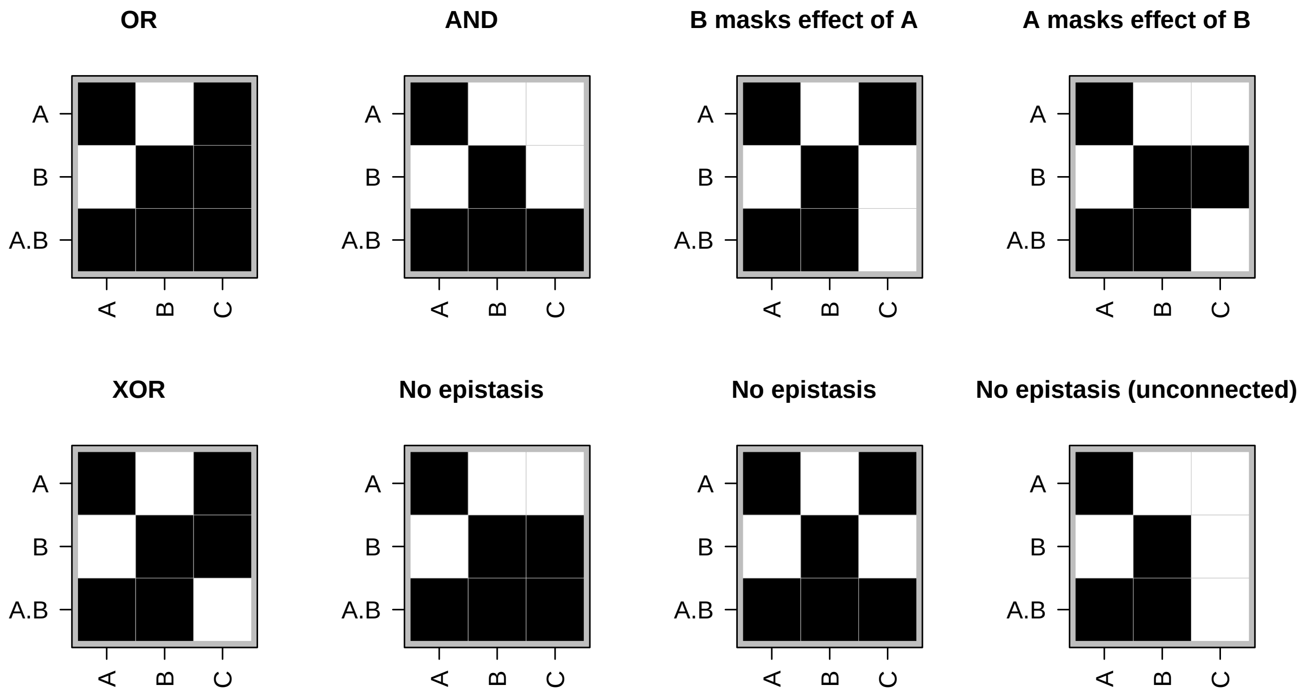

Figure D: Perfect binary effects matrix for each logic.

B-NEM is a method for general Boolean network inference based on multivariate read-out from combinatorial perturbation experiments. EpiNEM is specifically designed for knock-out screens including double and single knock-outs. Thus epiNEM is faster especially for large scale screens and more accurate, because it is designed for triples.

We boxplot running time, accuracy for the inferred edges, logical gates and expected data (truth table) for all five respectively two methods.

```r
data(sim)
```

```r
plot(sim)
```

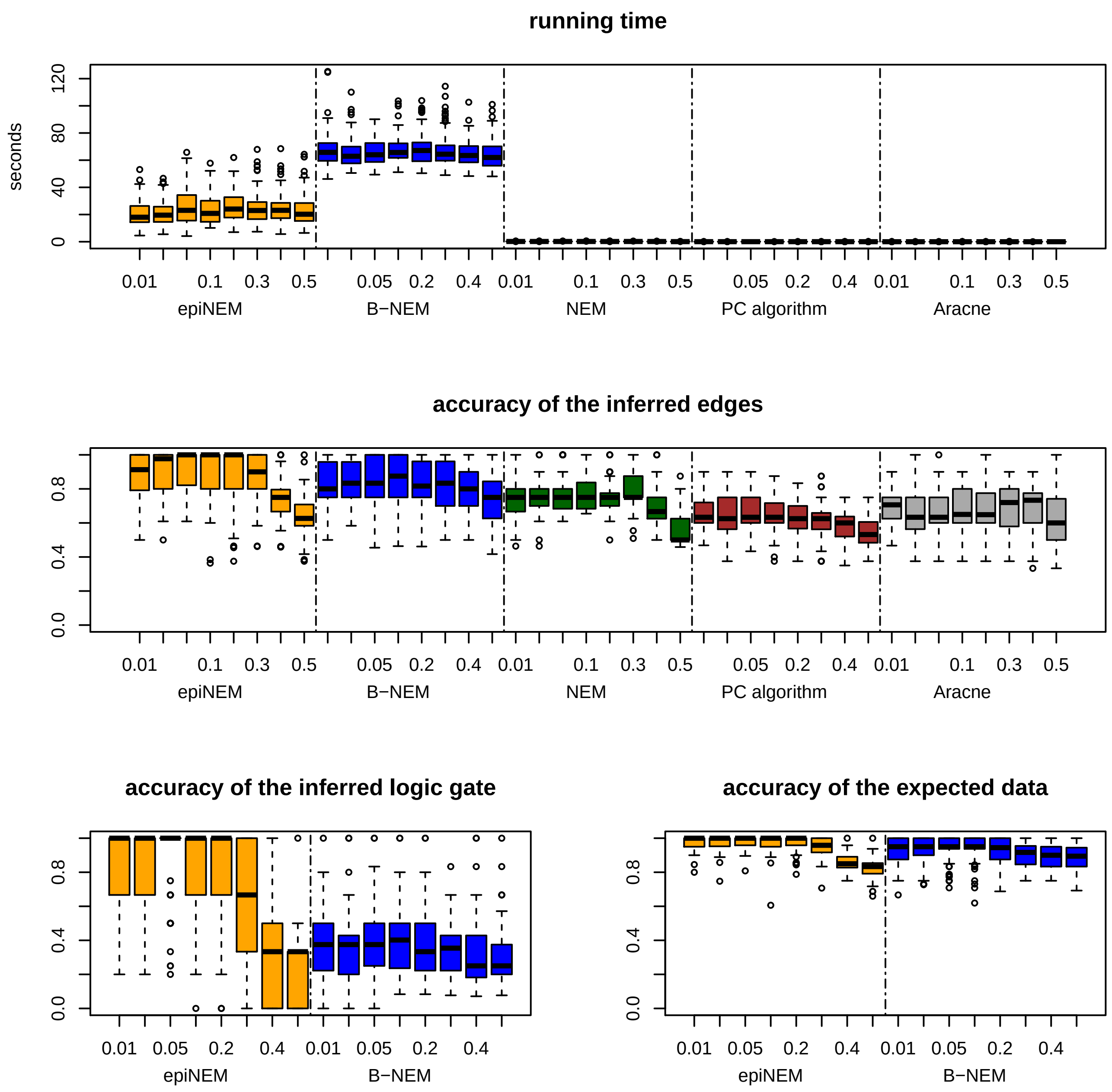

Figure E: Simulation results.

The plot shows that as expected B-NEM is the slowest followed by epiNEM, while NEM, ARACNE and the PC algorithm are much faster. However, the last three can not derive any logics in the network structure. epiNEM and B-NEM show almost equally high accuracy of inferred edges and truth tables (expected data). However, epiNEM reaches a much higher accuracy for inferred logic gates, which is due to the much higher degree of equivalent network structures in the case of B-NEM.

5 Yeast knock-out screens

In this section we analyse previously published yeast knock-out screens. The screens consist of gene expression data derived from double and single knock-out mutants. We use epiNEM on each double mutant combined with each single mutant.

5.1 Identification of significant modulators for pairs of double knock-outs

The first knock-out screen is from van Wageningen et al., 2010 and was created to investigate relationships between phosphorylation based pathways.

We applied epiNEM to all triples and infer logics and calculated the log-likelihood.

The following plot shows the global distribution of the inferred logical gates for all pairs. The single modulators are on the y-axis and the double knock-out pairs are on the x-axis.

```
data(wagscreen)

doubles <- wagscreen$doubles

dataWag <- wagscreen$dataWag

## clean up the results:

if (length(grep("fus3|ptp2.ptc2", wagscreen$doubles)) > 0) {
    wagscreen$doubles <- wagscreen$doubles[-grep("fus3|ptp2.ptc2",
                                                 wagscreen$doubles)]

    wagscreen$dataWag <- wagscreen$dataWag[, -grep("fus3|ptp2.ptc2",
                                                   colnames(wagscreen$dataWag))]

    wagscreen$ll <- wagscreen$ll[, -grep("fus3|ptp2.ptc2",
                                         colnames(wagscreen$ll))]

    wagscreen$logic <- wagscreen$logic[, -grep("fus3|ptp2.ptc2",
                                               colnames(wagscreen$logic))]
}

plot(wagscreen, xrot = 45, borderwidth = 0)
```

We can also plot the significant results for every explicit triple with log-likelihoods on the y-axis and ranked triples on the x-axis. The “ind” parameter, if set, selects a subset, i.e. double knock-out.

```
plot(wagscreen, global = FALSE, ind = 3, cexGene = 0.7, cexLegend = 0.9,
     off = 0.2)
```

The second knock-out screen by Sameith et al., 2015 was created to investigate growth based genetic interactions.

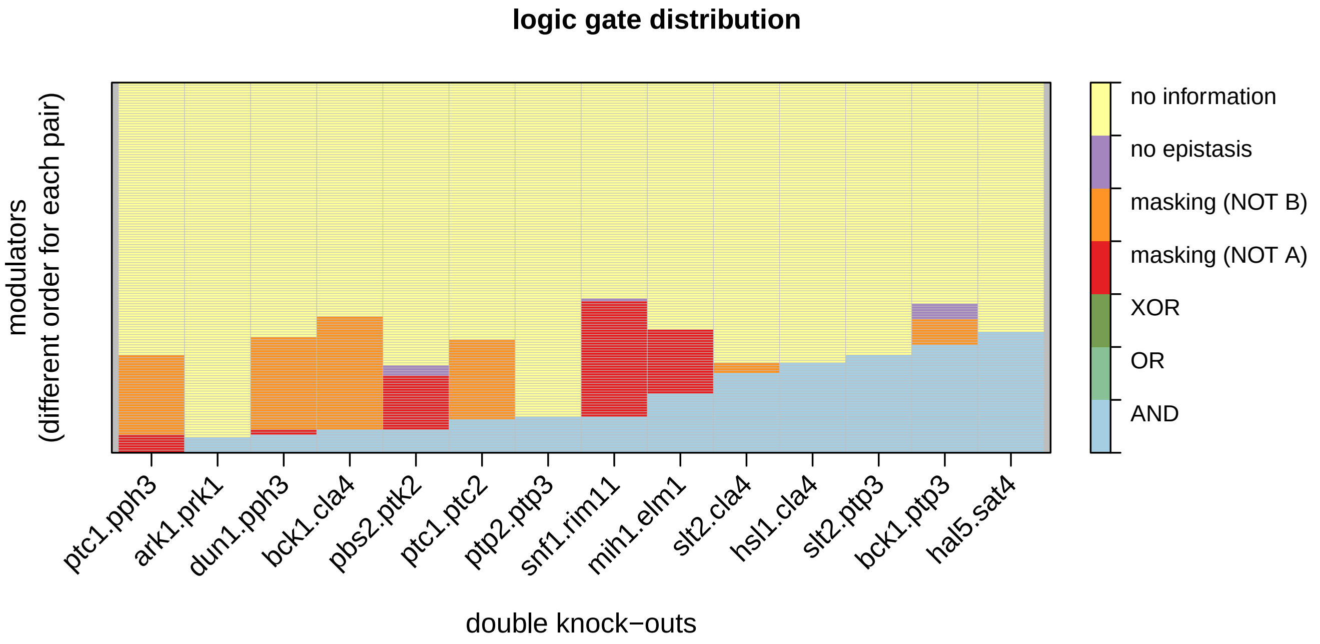

Figure F: Global results for the van Wageningen data set. See the help pages of plot.epiScreen() and HeatmapOP() for additional parameters.

Again, we plot the global distribution of logics followed by the explicit results for every identified modulator of one double knock-out.

```
data(samscreen)

doubles <- samscreen$doubles

dataSam <- samscreen$dataSam

plot(samscreen, xrot = 45, cexCol = 0.6, borderwidth = 0)
```

```
plot(samscreen, global = FALSE, ind = 23, cexGene = 0.7, cexLegend = 0.9,
     off = 0.2)
```

5.2 Validation of identified interactions

5.2.1 String-db interactions

We plot the densities of the string-db interaction scores of our identified modulators and the background density to visualize the significance of the interaction identified by epiNEM.

We plot the densities of the distributions of the background distribution (blue) and the scores of a pair from the triplets by epiNEM (pink). Additionally we calculate, if the difference between the two score distributions is significant (Mann-Whitney test).

We do this for both the van Wageningen et al., and the Sameith et al., data sets.

```
library(STRINGdb)

get_STRING_species(version="10", species_name=NULL)[26, ] # 4932
```

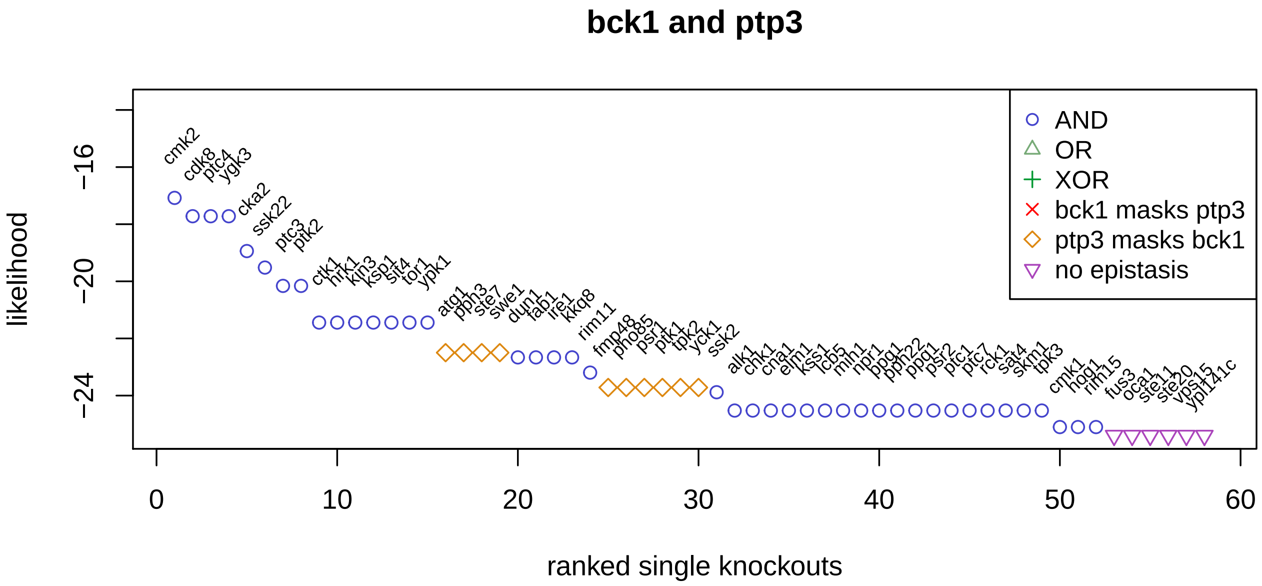

Figure G: Results for one double knock-out of the van Wageningen data set. See ?plot.epiScreen for further parameters.

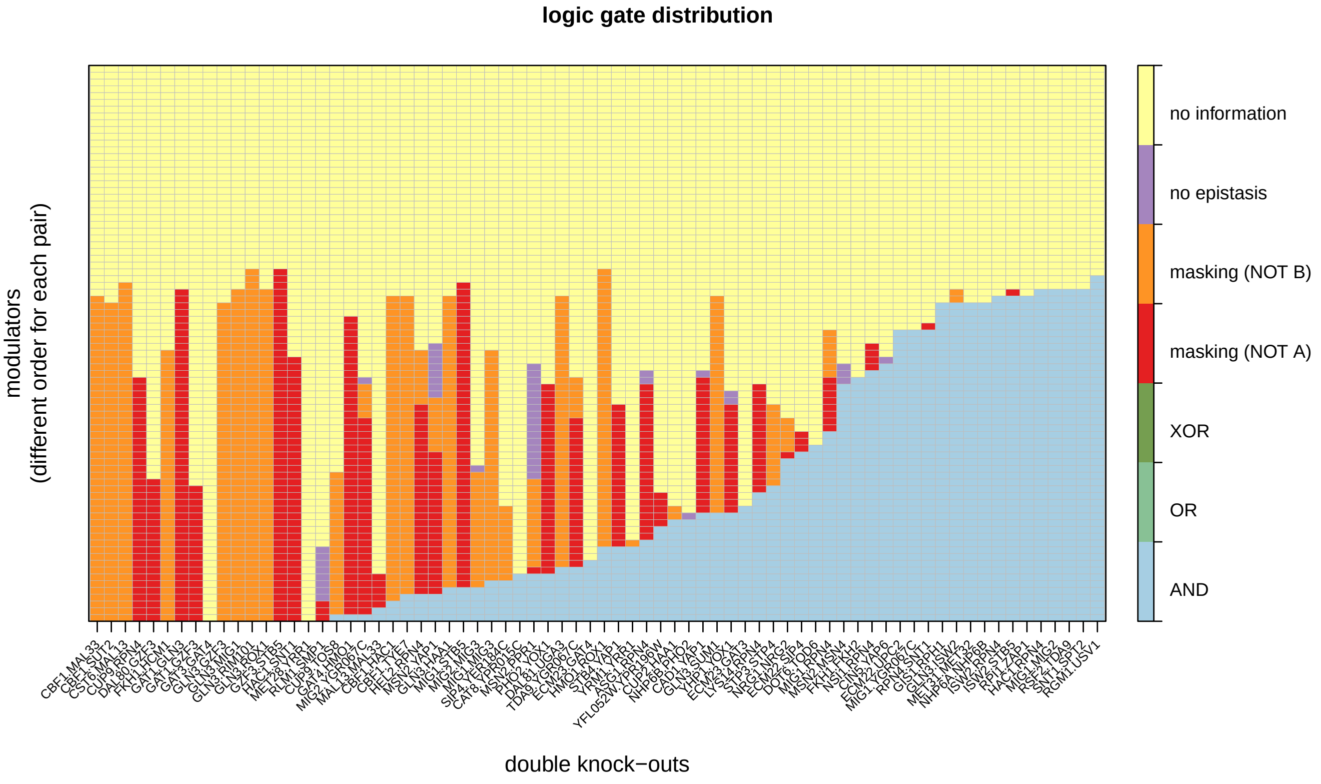

Figure H: Global result for the Sameith data set.

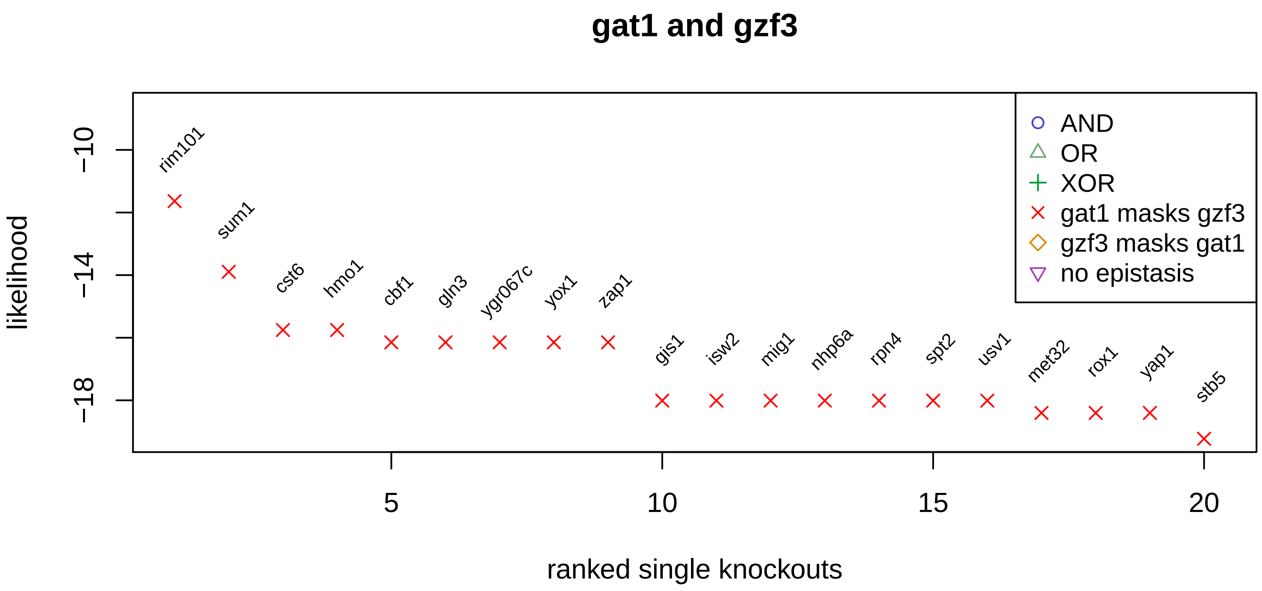

Figure I: Example for one knock-out of the Sameith data set.

```
##    species_id            official_name             compact_name   kingdom
## 26       4932 Saccharomyces cerevisiae Saccharomyces cerevisiae eukaryota
##    type
## 26 core

string_db <- STRINGdb$new( version="10", species=4932, score_threshold=0,
                          input_directory="")
data(wageningen_string)

string.scores <- wageningen_string$string.scores
string.names <- wageningen_string$string.names

tmp <- string_db$get_interactions(
    string_db$mp(unique(unlist(strsplit(colnames(dataWag), "\\.")))))

stsc <- unlist(string.scores)

denspval <- wilcox.test(stsc, unlist(tmp$combined_score),
                        alternative = "greater")$p.value

for (i in 100:1) {

    if (denspval < 10^(-i)) {

        denspval <- paste("< ", 10^(-i), sep = "")

    }

}
```

```
plot(density(stsc), col = "#00000000",
     ylim = c(0, max(c(max(density(stsc)$y),
                       max(density(unlist(tmp$combined_score))$y)))),
     main = paste("van Wageningen String-db interaction scores", sep = ""),
     xlab = "",
     cex.main = 1.5)
polygon(density(stsc), col = "#ff000066")

legend("topright", legend=paste("p-value", denspval, " "), cex = 1.5)

mtext = mtext("A", side = 3, line = 1, outer = FALSE, cex = 3, adj = 0,
              at = par("usr")[1] - (par("usr")[2]-par("usr")[1])*0.1)

lines(density(unlist(tmp$combined_score)), col = "#00000000")
polygon(density(unlist(tmp$combined_score)), col = "#00ffff66")
```

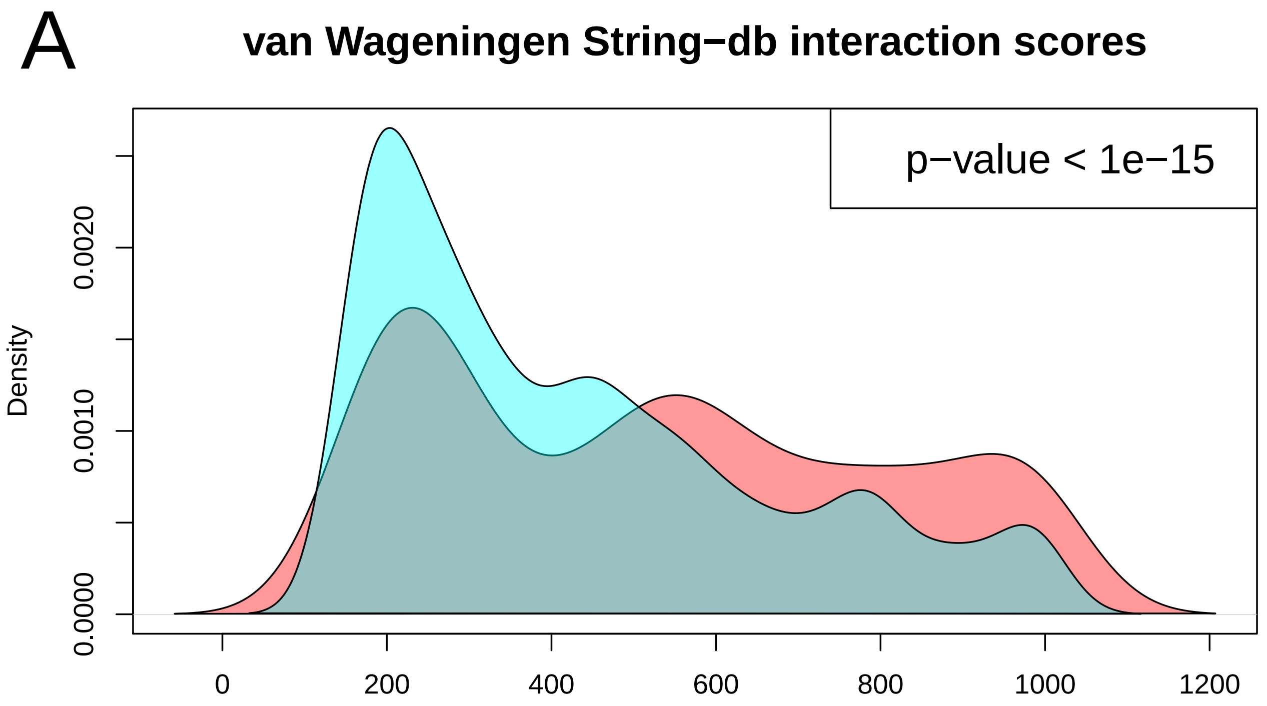

Figure J: Density of the string-db interaction scores (van Wageningen). Background (turqoise) and inferred by epiNEM (pink).

```
data(sameith_string)

string.scores2 <- sameith_string$string.scores2
string.names2 <- sameith_string$string.names2

tmp <- string_db$get_interactions(
    string_db$mp(unique(unlist(strsplit(colnames(dataSam), "\\.")))))
```

```
stsc <- unlist(string.scores2)

denspval <- wilcox.test(stsc, unlist(tmp$combined_score),
                        alternative = "greater")$p.value

for (i in 100:1) {

    if (denspval < 10^(-i)) {

        denspval <- paste("< ", 10^(-i), sep = "")

    }

}

plot(density(stsc), col = "#00000000",
     ylim = c(0, max(c(max(density(stsc)$y),
                       max(density(unlist(tmp$combined_score))$y)))),
     main = paste("Sameith String-db interaction scores", sp = ""),
     xlab = "",
     cex.main = 1.5)
polygon(density(stsc), col = "#ff000066")

legend("topright", legend=paste("p-value", denspval, " "), cex = 1.5)

mtext = mtext("B", side = 3, line = 1, outer = FALSE, cex = 3, adj = 0,
              at = par("usr")[1] - (par("usr")[2]-par("usr")[1])*0.1)

lines(density(unlist(tmp$combined_score)), col = "#00000000")
polygon(density(unlist(tmp$combined_score)), col = "#00ffff66")
```

5.2.2 Graph based GO similarity scores

We perform a similar analysis to the STRING interactions with a graph-based GO term similarity score (Wang et al., 2007) implemented in the R-package GOSemSim (Yu et al., 2010). This score is more flexible and allows us to calculate the similarity of GO terms between the parent double knock-out pair and the identified modulator instead of just pairwise interactions. For the average GO term similarity epiNEMs did not identify more significant interactions. However, the maximum similar GO term between parents and modulator is significantly higher on average than by random guessing (Fig. 14, 15).

```
data(wageningen_GO)

GOepi <- wageningen_GO$epi
GOall <- wageningen_GO$all

denspval <- wilcox.test(GOepi, GOall, alternative = "greater")$p.value

for (i in 100:1) {
    if (i <= 2) {
        for (j in 1:9) {
            if (denspval < j*10^(-i)) {
                denspval <- paste("< ", j*10^(-i), sep = "")
            }
```

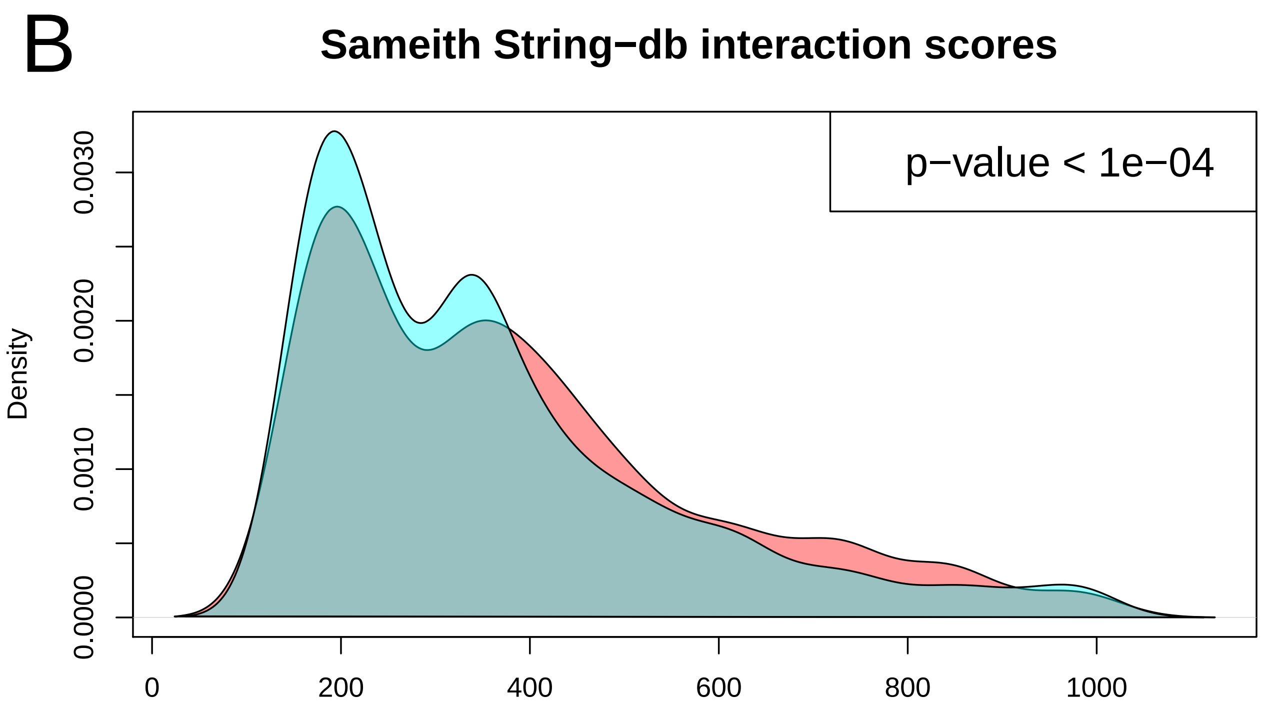

Figure K: Density of the string-db interaction scores (van Wageningen). Background (turqoise) and inferred by epiNEM (pink).

```
        }
    } else {
        if (denspval < 10^(-i)) {
            denspval <- paste("< ", 10^(-i), sep = "")
        }
    }
}

plot(density(GOepi), col = "#00000000",
     ylim = c(0, max(c(max(density(GOepi)$y),
                       max(density(unlist(GOall))$y)))),
     main = "van Wageningen Go similarity scores",
     xlab = "",
     cex.main = 1.5)
polygon(density(GOepi), col = "#ff000066")

legend("topleft", legend=paste("p-value", denspval, "   "), cex = 1.5)

mtext = mtext("C", side = 3, line = 1, outer = FALSE, cex = 3, adj = 0,
              at = par("usr")[1] - (par("usr")[2]-par("usr")[1])*0.1)

lines(density(unlist(GOall)), col = "#00000000")
polygon(density(unlist(GOall)), col = "#00ffff66")
```

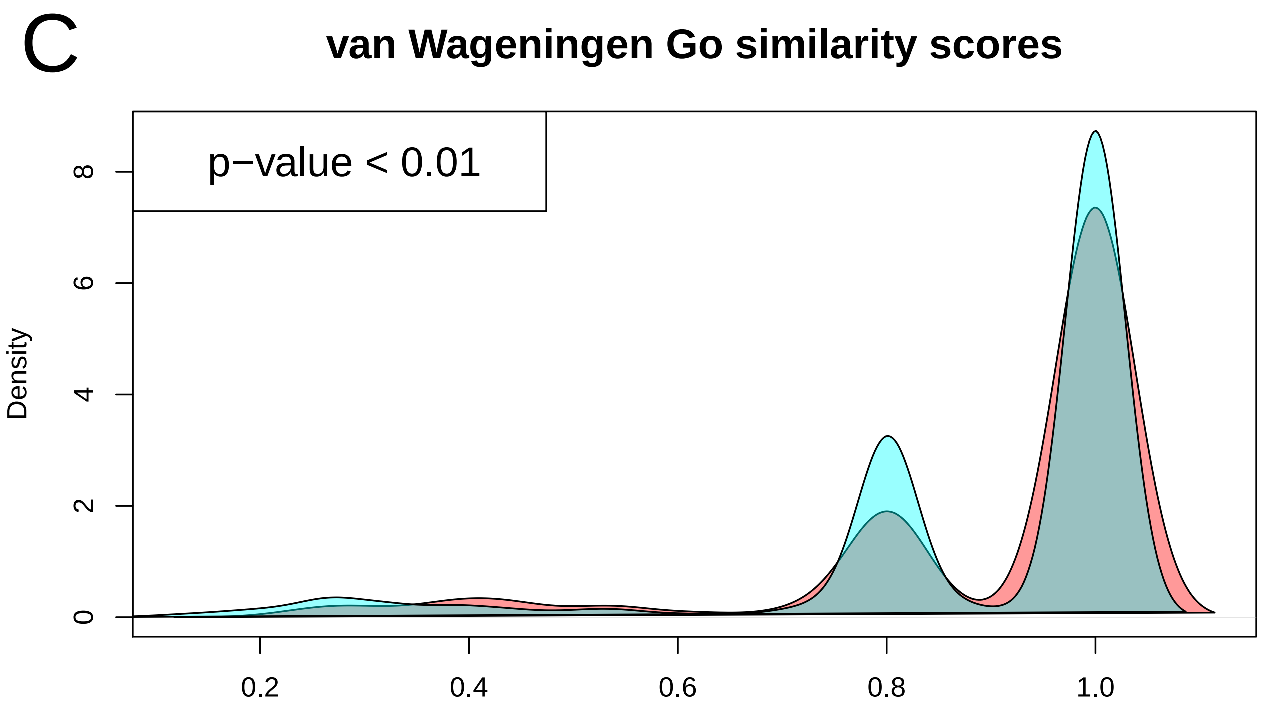

Figure L: Density plot for graph-based GO similarity score (van Wageningen).

```
data(sameith_GO)

GOepi2 <- sameith_GO$epi
GOall2 <- sameith_GO$all

denspval <- wilcox.test(GOepi2, GOall2, alternative = "greater")$p.value

for (i in 100:1) {
    if (i <= 2) {
        for (j in 1:9) {
            if (denspval < j*10^(-i)) {
                denspval <- paste("< ", j*10^(-i), sep = "")
            }
        }
    } else {
        if (denspval < 10^(-i)) {
            denspval <- paste("< ", 10^(-i), sep = "")
        }
    }
}

plot(density(GOepi2), col = "#00000000",
     ylim = c(0, max(c(max(density(GOepi2)$y),
                       max(density(unlist(GOall2))$y)))),
```

```
     main = "Sameith Go similarity scores",
     xlab = "",
     cex.main = 1.5)
polygon(density(GOepi2), col = "#ff000066")

legend("topleft", legend=paste("p-value", denspval, "   "), cex = 1.5)

mtext = mtext("D", side = 3, line = 1, outer = FALSE, cex = 3, adj = 0,
              at = par("usr")[1] - (par("usr")[2]-par("usr")[1])*0.1)

lines(density(unlist(GOall2)), col = "#00000000")
polygon(density(unlist(GOall2)), col = "#00ffff66")
```

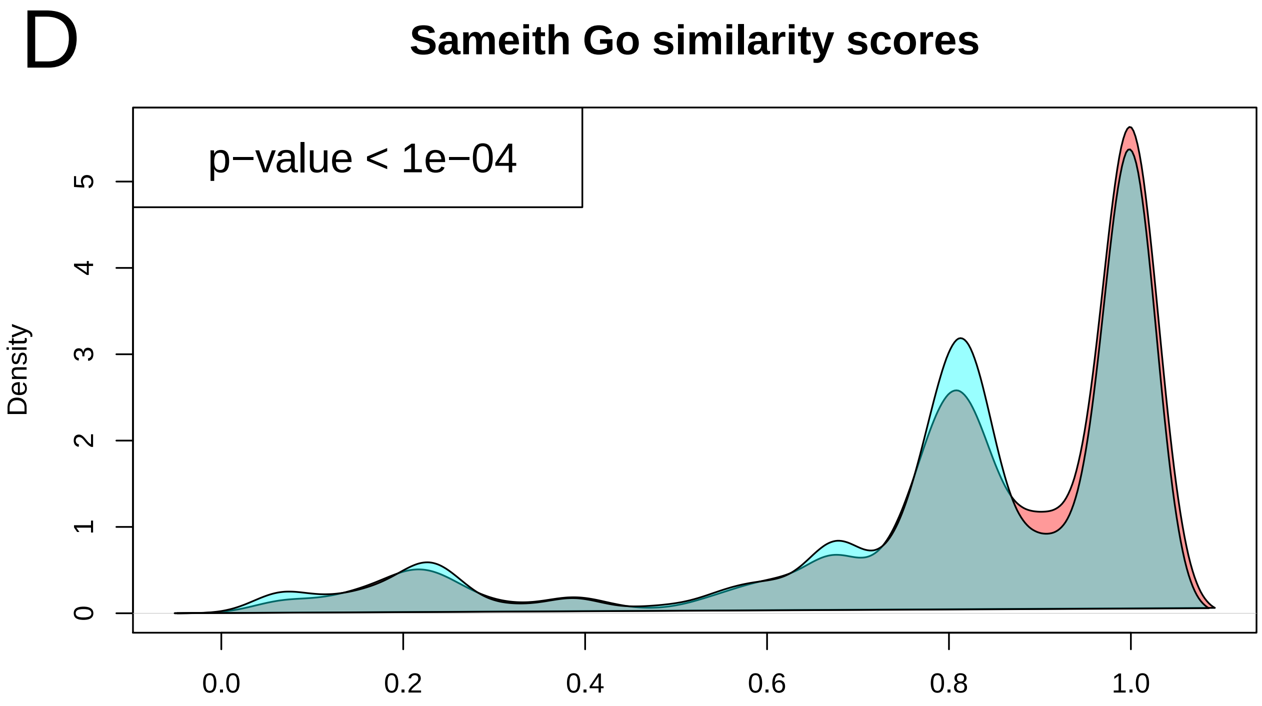

Figure M: Density plot for graph-based GO similarity score (Sameith).

5.3 Enrichment analysis

5.3.1 Enrichment of identified sets of modulators for each double knock-out

We further analyze the set of identified modulators for each pair by KEGG pathway enrichment. Fig. 14 and 15 show heatmaps of false discovery rates. Knock-out pairs are in the columns and enriched pathways in the rows. Red denotes no enrichment. The most significant pathways are meiosis, cell cycle and MAPK signaling, which are enriched for almost all pairs in both data sets. Interestingly, these are the only pathways we find for the Sameith *et al.*, data set. For the van Wageningen *et al.*, data set we find several more pathways, which are only enriched in a subset of knock-out pairs, e.g. glycerophospholipid metabolism is highly enriched for the *ptp2,ptp3* and *hal5,sat4* double mutants only.

```
data(wageningen_GO)

golist <- wageningen_GO$golist

goterms <- character()

for (i in 1:length(golist)) {

    if (i %in% c(5,8)) { next() }

    goterms <- c(goterms,
                 golist[[i]]$term_description[which(golist[[i]]$pvalue_fdr
                                                    < 1)])

}

gomat <- matrix(NA, length(unique(goterms)), ncol(wagscreen$ll))

rownames(gomat) <- sort(unique(goterms))
colnames(gomat) <- colnames(wagscreen$ll)

for (i in 1:ncol(wagscreen$ll)) {

    gotmp <- golist[[i]]
    gotmp <- gotmp[order(gotmp$term_description), ]

    gomat[which(rownames(gomat) %in% golist[[i]]$term_description), i] <-
        golist[[i]][which(golist[[i]]$term_description %in% rownames(gomat)), 4]

}

if (nrow(gomat) > 20) {
    rownames(gomat) <- NULL
}

HeatmapOP(gomat,
          bordercol = "transparent",
          main = "", sub = "",
          xrot = 45, col = "RdYlBu", breaks = 100)
```

```
data(sameith_GO)

golist2 <- sameith_GO$golist

goterms <- character()

for (i in 1:length(golist2)) {

    goterms <-
        c(goterms,
          golist2[[i]]$term_description[which(golist2[[i]]$pvalue_fdr < 0.1)])

}
```

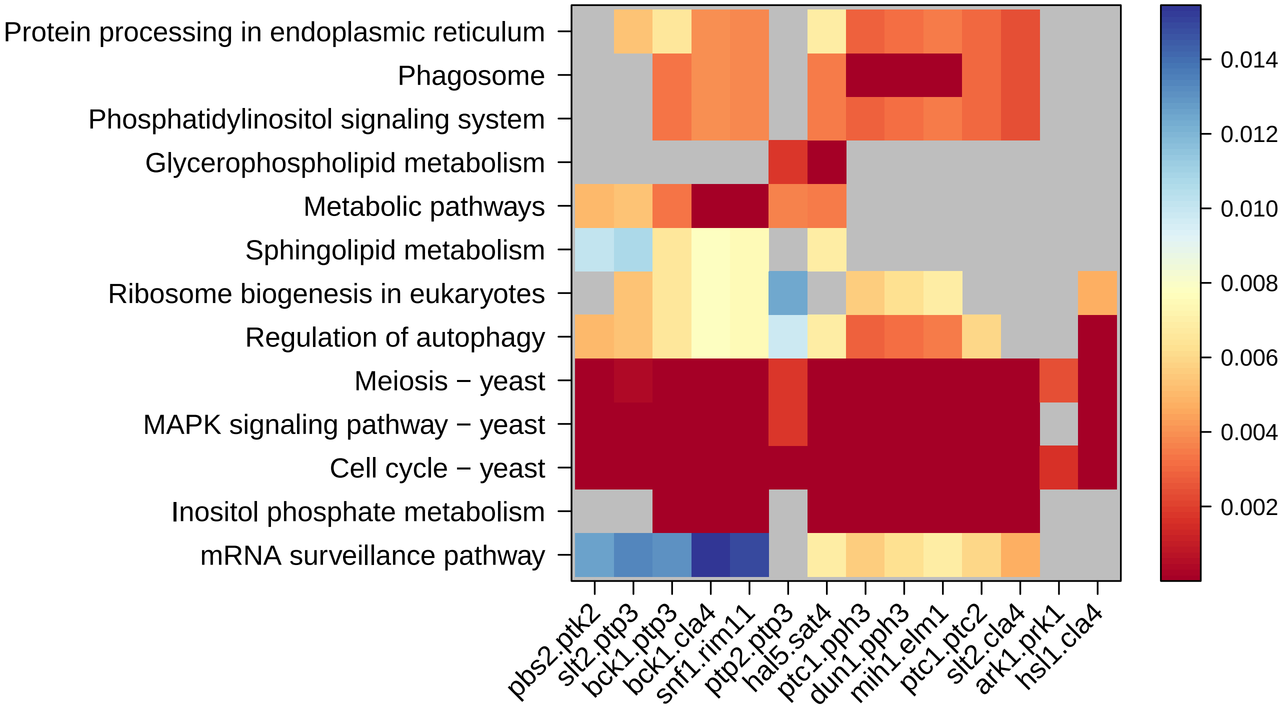

Figure N: Enrichment of van Wageningen modulators by KEGG pathways. Colors refer to false discovery rates. NAs are colored in grey.

```
gomat <- matrix(NA, length(unique(goterms)), ncol(samscreen$ll))

rownames(gomat) <- sort(unique(goterms))
colnames(gomat) <- colnames(samscreen$ll)

for (i in 1:ncol(samscreen$ll)) {

    gotmp <- golist2[[i]]
    gotmp <- gotmp[order(gotmp$term_description), ]

    gomat[which(rownames(gomat) %in% golist2[[i]]$term_description), i] <-
        golist2[[i]][which(golist2[[i]]$term_description %in%
                           rownames(gomat)), 4]

}

if (nrow(gomat) > 20) {
    rownames(gomat) <- NULL
}

colnames(gomat) <- tolower(colnames(gomat))

HeatmapOP(gomat,
```

```
          bordercol = "transparent",
          main = "", sub = "",
          xrot = 45, cexCol = 0.5, col = "RdYlBu", breaks = 100)
```

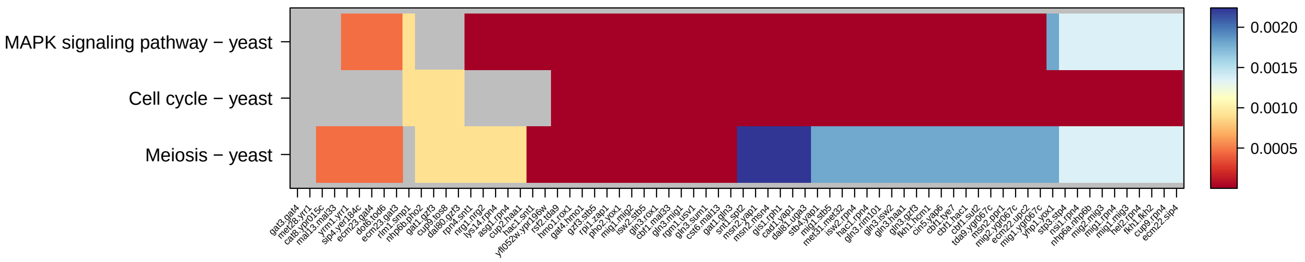

Figure O: Enrichment of Sameith modulators by KEGG pathways. Colors refer to false discovery rates. NAs are colored in grey.

5.3.2 Enrichment of effect reporters for each identified modulator

EpiNEM calculates the a posteriori attachment for each effect reporter. Thus, for each significant modulator and corresponding double mutant pair we can identify genes directly regulated by that modulator. As a summary of enriched GO terms, Fig. 16 and 17 show enriched pathways (rows) for effect reporters of the respective modulators (columns). The false discovery rates are averaged over all double mutant pairs which identified the modulator as significant. Interestingly, we find the previously significant pathways like Meiosis and MAPK signaling, but at a lower rate than before (Fig. 16). However, overall all modulators in both data sets show strong enrichment in a similar set of pathways.

```
gos <- unique(wageningen_GO$gos)

egenego <- wageningen_GO$egenego

gomat <- array(NA, c(length(gos), nrow(wagscreen$ll), ncol(wagscreen$ll)))

rownames(gomat) <- sort(gos)

colnames(gomat) <- rownames(wagscreen$ll)

dimnames(gomat)[[3]] <- colnames(wagscreen$ll)

for (i in 1:length(wagscreen$targets)) {
    if (length(wagscreen$targets[[i]]) == 0) { next() }
    for (j in 1:length(wagscreen$targets[[i]])) {
        if (dim(egenego[[i]][[j]])[1] > 0) {
            gomat[which(rownames(gomat) %in%
                        egenego[[i]][[j]]$term_description),
                  which(dimnames(gomat)[[2]] %in%
                        names(wagscreen$targets[[i]])[j]), i] <-
                egenego[[i]][[j]]$pvalue_fdr[
                                  order(egenego[[i]][[j]]$term_description)]
        }
    }
}
```

```
gomat <- apply(gomat, c(1,2), mean, na.rm = TRUE)

gomat <- gomat[order(apply(gomat, 1, function(x)
    return(sum(is.na(x) == FALSE)))), ]

gomat <- gomat[, rev(order(apply(gomat, 2, function(x)
    return(sum(is.na(x) == FALSE)))))]

gomat <- gomat[, which(apply(gomat, 2,
                             function(x) return(any(is.na(x) == FALSE))))]

HeatmapOP(gomat, xrot = 45, Colv = FALSE, Rowv = FALSE,
          col = "RdYlBu", main = "", sub = "", breaks = 100)
```

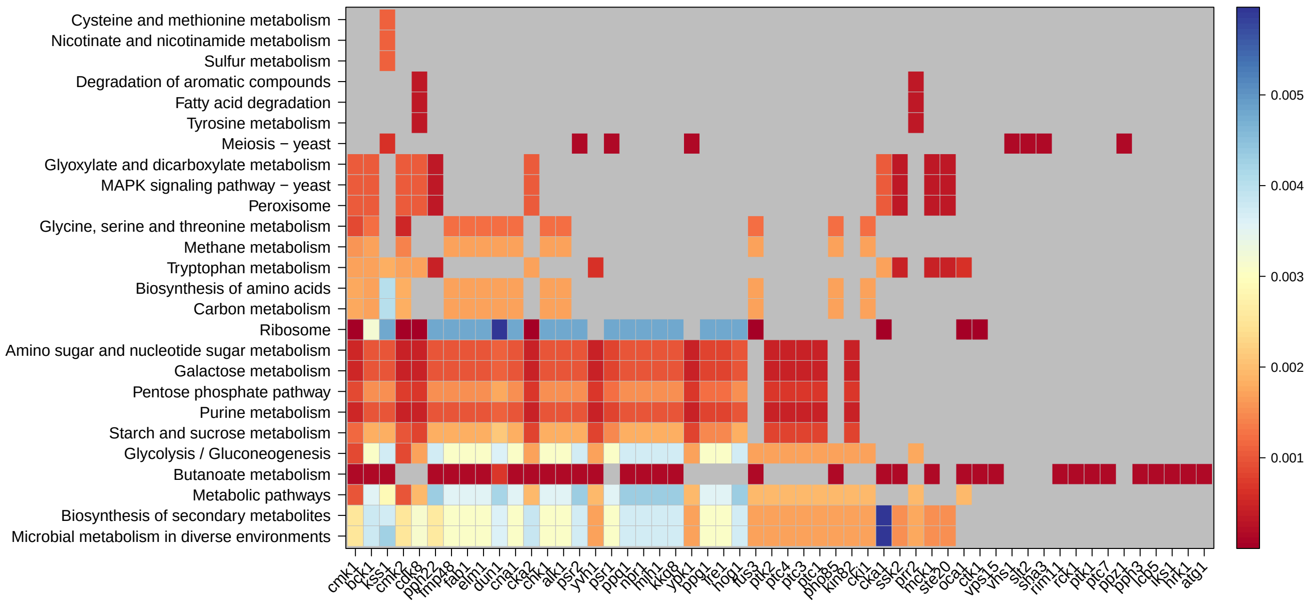

Figure P: Effect reporter KEGG pathway enrichment (van Wageningen). Colors refer to false discovery rates. NAs are colored in grey.

```
gos2 <- unique(sameith_GO$gos)

egenego2 <- sameith_GO$egenego

gomat <- array(NA, c(length(gos2), nrow(samscreen$ll), ncol(samscreen$ll)))

rownames(gomat) <- sort(gos2)

colnames(gomat) <- rownames(samscreen$ll)

dimnames(gomat)[[3]] <- colnames(samscreen$ll)

for (i in 1:length(samscreen$targets)) {
    if (length(samscreen$targets[[i]]) == 0) { next() }
```

```
    for (j in 1:length(samscreen$targets[[i]])) {
        if (dim(egenego2[[i]][[j]])[1] > 0) {
            gomat[which(rownames(gomat) %in%
                        egenego2[[i]][[j]]$term_description),
                  which(dimnames(gomat)[[2]] %in%
                        names(samscreen$targets[[i]])[j]), i] <-
                egenego2[[i]][[j]]$pvalue_fdr[
                                      order(
                                          egenego2[[i]][[j]]$term_description)]
        }
    }
}

gomat <- apply(gomat, c(1,2), mean, na.rm = TRUE)

gomat <- gomat[order(apply(gomat, 1, function(x)
    return(sum(is.na(x) == FALSE)))), ]

gomat <- gomat[, rev(order(apply(gomat, 2, function(x)
    return(sum(is.na(x) == FALSE)))))]

gomat <- gomat[, which(apply(gomat, 2,
                             function(x) return(any(is.na(x) == FALSE))))]

colnames(gomat) <- tolower(colnames(gomat))

HeatmapOP(gomat, xrot = 45, Colv = FALSE, Rowv = FALSE,
          col = "RdYlBu", main = "", sub = "", breaks = 100)
```

6 Creation of data objects

In this section we show the R code, which was used to create the simulation and application results.

```
###### simulation:

## install_github("MartinFXP/B-NEM"); library(bnem)

library(nem)

library(minet)

library(pcalg)

runs <- 100

noiselvls <- c(0.01, 0.025, 0.05, 0.1, 0.2, 0.3, 0.4, 0.5)

random <- list(FPrate = 0.1, FNrate = noiselvls,
               single = 4, double = 1, reporters = 100, replicates = 3)

do <- c("n", "p", "a", "e", "b")
```

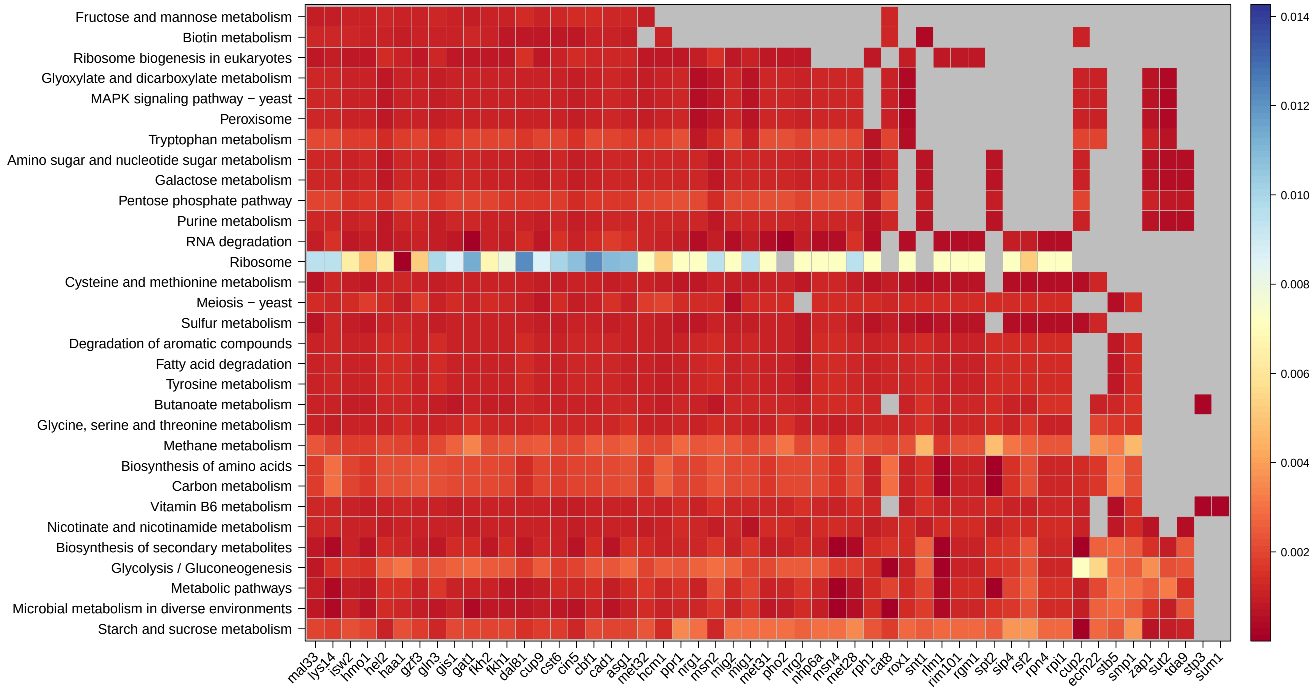

Figure Q: Effect reporter KEGG pathway enrichment (Sameith). Colors refer to false discovery rates. NAs are colored in grey.

```
maxTime <- FALSE

forcelogic <- TRUE

epinemsearch <- "greedy"

nIterations <- 3

bnemsearch <- "genetic"

simresults <- SimEpiNEM(runs, do, random, maxTime, forcelogic,
epinemsearch, bnemsearch, nIterations)

sim <- simresults

###### yeast van Wageningen et al.:

file <- paste("http://www.holstegelab.nl/",
              "publications/sv/signaling_redundancy/downloads/DataS1.txt",
              sep = "")

data <- read.delim(file)

dataM <- data[-(1), (1+(1:(324/2))*2)]
```

```
dataP <- data[-(1), (2+(1:(324/2))*2)]

data[, 2] <- as.character(data[, 2])

rndup <- which(duplicated(data[, 2]) == TRUE)

data[rndup, 2] <- paste(data[rndup, 2], "_dup", sep = "")

rownames(dataM) <- rownames(dataP) <- data[2:nrow(data), 2]

dataM <- dataM[-1, ]

dataP <- dataP[-1, ]

dataM <- apply(dataM, c(1,2), as.numeric)

dataP <- apply(dataP, c(1,2), as.numeric)

dataBin <- dataM

sig <- 0.05

cutoff <- log2(1.7)

dataBin[which(dataP < sig & dataP > 0 & abs(dataM) >= cutoff)] <- 1

dataBin[which(dataP >= sig | dataP == 0 | abs(dataM) < cutoff)] <- 0

dataBin <- dataBin[-which(apply(dataBin, 1, max) == 0), ]

dataBinWag <- dataBin

colnames(dataBin) <- gsub(".del.vs..wt", "", colnames(dataBin))

colnames(dataBin) <- gsub(".del", "", colnames(dataBin))

doubles <- colnames(dataBin)[grep("\\.", colnames(dataBin))]

if (length(grep("vs", doubles)) > 0) {
    doubles <- sort(doubles[-grep("vs", doubles)])
} else { doubles <- sort(doubles) }

doubles.genes <- unique(unlist(strsplit(doubles, "\\.")))

if (length(grep("\\.", colnames(dataBin))) > 0) {
    singles <- colnames(dataBin)[-grep("\\.", colnames(dataBin))]
} else { singles <- sort(singles) }

singles <- unique(sort(singles))

wagscreen <- epiScreen(dataBin[, -grep("fus3\\.|ptp2.ptc2", colnames(dataBin))])

wagscreen$dataWag <- dataBin[, -grep("fus3.|ptp2.ptc2", colnames(dataBin))]
```

```
###### yeast Sameith et al.:

file <- paste("http://www.holstegelab.nl/",
              "publications/GSTF_geneticinteractions/",
              "downloads/del_mutants_limma.txt", sep = "")

data <- read.delim(file)

data <- apply(data, c(1,2), as.character)

dataM <- data[-1, which(data[1, ] %in% "M")]

dataM <- apply(dataM, c(1,2), as.numeric)

dataP <- data[-1, which(data[1, ] %in% "p.value")]

dataP <- apply(dataP, c(1,2), as.numeric)

rownames(dataM) <- rownames(dataP) <- data[2:nrow(data), 1]

dataBin <- dataM

sig <- 0.01

cutoff <- log2(1.5)

dataBin[which(dataP < sig & dataP > 0 & abs(dataM) >= cutoff)] <- 1

dataBin[which(dataP >= sig | dataP == 0 | abs(dataM) < cutoff)] <- 0

dataBin <- dataBin[-which(apply(dataBin, 1, max) == 0), ]

colnames(dataBin) <- gsub("\\.\\.\\.", "\\.", colnames(dataBin))

doubles <- colnames(dataBin)[grep("\\.", colnames(dataBin))]

if (length(grep("vs", doubles)) > 0) {
    doubles <- sort(doubles[-grep("vs", doubles)])
} else { doubles <- sort(doubles) }

doubles.genes <- unique(unlist(strsplit(doubles, "\\.")))

if (length(grep("\\.", colnames(dataBin))) > 0) {
    singles <- colnames(dataBin)[-grep("\\.", colnames(dataBin))]
} else { singles <- sort(singles) }

singles <- unique(sort(singles))

samscreen <- epiScreen(dataBin)

samscreen$dataSam <- dataBin

## String-db interaction scores:
```

```
library(STRINGdb)

get_STRING_species(version="10", species_name=NULL)[26, ] # 4932

string_db <- STRINGdb$new( version="10", species=4932, score_threshold=0,
                           input_directory="")

llmat <- wagscreen$ll

logicmat <- wagscreen$logic

string.scores <- list()

string.names <- character()

for (i in 1:ncol(llmat)) {
    if (sum(!(llmat[, i] %in% c(0,-Inf))) > 0) {
        top30 <- llmat[, i]
        top30[which(top30 == 0)] <- -Inf
        top30 <- top30[which(!(llmat[, i] %in% c(0,-Inf)))]
        top30 <- top30[order(top30,decreasing = TRUE)[1:min(30, sum(!(llmat[, i]
            %in% c(0,-Inf))))]]

        doubles <- unlist(strsplit(colnames(llmat)[i], "\\."))

        for (j in names(top30)) {
            tmp <- string_db$get_interactions(string_db$mp(c(doubles[1], j)))
            string.scores <- c(string.scores, tmp$combined_score)
            string.names <- c(string.names, paste(sort(c(doubles[1], j)),
                                                  collapse = "_"))
            tmp <- string_db$get_interactions(string_db$mp(c(doubles[2], j)))
            string.scores <- c(string.scores, tmp$combined_score)
            string.names <- c(string.names, paste(sort(c(doubles[2], j)),
                                                  collapse = "_"))
        }

    } else {
        next()
    }
}


llmat <- samscreen$ll

logicmat <- samscreen$logic

string.scores2 <- list()

string.names2 <- character()

for (i in 1:ncol(llmat)) {

    if (sum(!(llmat[, i] %in% c(0,-Inf))) > 0) {
```

```
        top30 <- llmat[, i]
        top30[which(top30 == 0)] <- -Inf
        top30 <- top30[which(!(llmat[, i] %in% c(0,-Inf)))]
        top30 <- top30[order(top30, decreasing = TRUE)
                       [1:min(30, sum(!(llmat[, i] %in% c(0,-Inf))))]]

        doubles <- unlist(strsplit(colnames(llmat)[i], "\\."))

        for (j in names(top30)) {
            tmp <- string_db$get_interactions(string_db$mp(c(doubles[1], j)))
            string.scores2 <- c(string.scores2, tmp$combined_score)
            string.names2 <- c(string.names2, paste(sort(c(doubles[1], j)),
                                                    collapse = "_"))
            tmp <- string_db$get_interactions(string_db$mp(c(doubles[2], j)))
            string.scores2 <- c(string.scores2, tmp$combined_score)
            string.names2 <- c(string.names2, paste(sort(c(doubles[2], j)),
                                                    collapse = "_"))
        }

    } else {
        next()
    }

}

## graph based GO similarity scores:

library(GOSemSim)
library(AnnotationHub)
library(org.Sc.sgd.db)

ystGO <- godata("org.Sc.sgd.db", ont = "BP",
                keytype = keytypes(org.Sc.sgd.db)[11], computeIC = FALSE)

## van Wageningen et al.:

GOepi <- numeric()

for (i in 1:ncol(wagscreen$ll)) {
    if (i %in% grep("fus3|ptp2.ptc2", colnames(wagscreen$ll))) { next() }
    pair <- toupper(unlist(strsplit(colnames(wagscreen$ll)[i], "\\.")))
    for (j in which(!is.infinite(wagscreen$ll[, i]) == TRUE &
                    wagscreen$ll[, i] != 0)) {
        tmp <- clusterSim(pair, toupper(rownames(wagscreen$ll)[j]),
                          semData = ystGO, combine = "max")
        if (!is.na(tmp[1])) {
            GOepi <- c(GOepi, tmp)
        }
    }
}

GOall <- numeric()
```

```
for (i in colnames(wagscreen$ll)) {
    pair <- toupper(unlist(strsplit(i, "\\.")))
    for (j in rownames(wagscreen$ll)) {
        tmp <- clusterSim(pair, toupper(j), semData = ystGO, combine = "max")
        if (!is.na(tmp[1])) {
            GOall <- c(GOall, tmp)
        }
    }
}

## Sameith et al.:

GOepi2 <- numeric()

for (i in 1:ncol(samscreen$ll)) {
    if (i %in% grep("fus3|ptp2.ptc2", colnames(samscreen$ll))) { next() }
    pair <- toupper(unlist(strsplit(colnames(samscreen$ll)[i], "\\.")))
    for (j in which(!is.infinite(samscreen$ll[, i]) == TRUE &
                    samscreen$ll[, i] != 0)) {
        tmp <- clusterSim(pair, toupper(rownames(samscreen$ll)[j]),
                       semData = ystGO, combine = "max")
        if (!is.na(tmp[1])) {
            GOepi2 <- c(GOepi2, tmp)
        }
    }
}

GOall2 <- numeric()

for (i in colnames(samscreen$ll)) {
    pair <- toupper(unlist(strsplit(i, "\\.")))
    for (j in rownames(samscreen$ll)) {
        tmp <- clusterSim(pair, toupper(j), semData = ystGO, combine = "max")
        if (!is.na(tmp[1])) {
            GOall2 <- c(GOall2, tmp)
        }
    }
}

###### Go enrichment analysis:

## van Wageningen et al.:

string_db$set_background(
    string_db$mp(unique(c(unlist(strsplit(colnames(wagscreen$ll), "\\.")),
                          rownames(wagscreen$ll)))))

golist <- list()

for (i in 1:ncol(wagscreen$ll)) {
    golist[[i]] <- string_db$get_enrichment(string_db$mp(unique(
        c(unlist(strsplit(colnames(wagscreen$ll)[i], "\\.")),
          rownames(wagscreen$ll)[which(!(wagscreen$logic[, i] %in%
```

```
                                        c("NOINFO", "NOEPI"))]]))),
        category = "KEGG", methodMT = "fdr", iea = TRUE)
}

string_db$set_background(string_db$mp(rownames(wagscreen$dataWag)))

egenego <- list()

gos <- character()

for (i in 1:length(wagscreen$targets)) {
    egenego[[i]] <- list()
    if (length(wagscreen$targets[[i]]) == 0) { next() }
    for (j in 1:length(wagscreen$targets[[i]])) {
        egenego[[i]][[j]] <- string_db$get_enrichment(
            string_db$mp(wagscreen$targets[[i]][[j]]),
            category = "KEGG", methodMT = "fdr", iea = TRUE)
        if (dim(egenego[[i]][[j]])[1] > 0) {
            gos <- c(gos, egenego[[i]][[j]]$term_description)
        }
    }
}
## Sameith et al.:

string_db$set_background(string_db$mp(unique(c(unlist(
    strsplit(colnames(samscreen$ll), "\\.")), rownames(samscreen$ll)))))

golist2 <- list()

for (i in 1:ncol(samscreen$ll)) {
    golist2[[i]] <- string_db$get_enrichment(string_db$mp(
        unique(c(unlist(strsplit(colnames(samscreen$ll)[i], "\\.")),
                 rownames(samscreen$ll)
                 [which(!(samscreen$logic[, i] %in% c("NOINFO", "NOEPI")))]))),
        category = "KEGG", methodMT = "fdr", iea = TRUE)
}

string_db$set_background(string_db$mp(rownames(samscreen$dataWag)))

egenego2 <- list()

gos2 <- character()

for (i in 1:length(samscreen$targets)) {
    egenego2[[i]] <- list()
    if (length(samscreen$targets[[i]]) == 0) { next() }
    for (j in 1:length(samscreen$targets[[i]])) {
        egenego2[[i]][[j]] <- string_db$get_enrichment(
            string_db$mp(samscreen$targets[[i]][[j]]),
            category = "KEGG", methodMT = "fdr", iea = TRUE)
        if (dim(egenego2[[i]][[j]])[1] > 0) {
            gos2 <- c(gos2, egenego2[[i]][[j]]$term_description)
        }
```

```
    }
}
```

7 Session information

```
sessionInfo()
## R Under development (unstable) (2017-01-11 r71964)
## Platform: x86_64-apple-darwin13.4.0 (64-bit)
## Running under: OS X El Capitan 10.11.6
##
## locale:
## [1] C/UTF-8/C/C/C/C
##
## attached base packages:
## [1] stats     graphics  grDevices utils     datasets  methods   base
##
## other attached packages:
## [1] STRINGdb_1.15.0  epiNEM_0.99.51   BiocStyle_2.3.31
##
## loaded via a namespace (and not attached):
##  [1] gtools_3.5.0        statmod_1.4.29      lattice_0.20-34
##  [4] htmltools_0.3.5     stats4_3.4.0        fastICA_1.2-0
##  [7] hash_2.2.6          yaml_2.1.14         gmp_0.5-13
## [10] chron_2.3-50        RBGL_1.51.0         XML_3.98-1.5
## [13] BoolNet_2.1.3       e1071_1.6-8         DBI_0.6
## [16] Rgraphviz_2.19.0    BiocGenerics_0.21.3 RColorBrewer_1.1-2
## [19] gsubfn_0.6-6        nem_2.49.0          plyr_1.8.4
## [22] robustbase_0.92-7   stringr_1.2.0       pcalg_2.4-5
## [25] bdsmatrix_1.3-2     caTools_1.17.1      memoise_1.0.0
## [28] evaluate_0.10       ggm_2.3             latticeExtra_0.6-28
## [31] knitr_1.15.1        parallel_3.4.0      class_7.3-14
## [34] DEoptimR_1.0-8      highr_0.6           proto_1.0.0
## [37] Rcpp_0.12.10        KernSmooth_2.23-15  corpcor_1.6.8
## [40] backports_1.0.5     limma_3.31.19       gdata_2.17.0
## [43] plotrix_3.6-4       graph_1.53.0        abind_1.4-5
## [46] gplots_3.0.1        png_0.1-7           digest_0.6.12
## [49] stringi_1.1.2       grid_3.4.0          rprojroot_1.2
## [52] clue_0.3-53         tools_3.4.0         bitops_1.0-6
## [55] sqldf_0.4-10        magrittr_1.5        RSQLite_1.1-2
## [58] RCurl_1.95-4.8      cluster_2.0.6       minet_3.33.0
## [61] rmarkdown_1.3.9004  boot_1.3-18         sfsmisc_1.1-0
## [64] igraph_1.0.1        compiler_3.4.0
```

8 References

Martin Pirkl, Madeline Diekmann, Marlies van der Wees, Niko Beerenwinkel, Holger Fröhlich, Florian Markowetz. Inferring Modulators of Genetic Interactions with Epistatic Nested Effects Models. Under review. 2017.

Robinson MD, McCarthy DJ, Smyth GK. edgeR: a Bioconductor package for differential expression analysis of digital gene expression data. Bioinformatics. 2010;26(1):139-140. doi:10.1093/bioinformatics/btp616.

Margolin AA, Nemenman I, Basso K, Wiggins C, Stolovitzky G, {Dalla Favera} R, et al. ARACNE: an algorithm for the reconstruction of gene regulatory networks in a mammalian cellular context. BMC Bioinformatics. 2006;7 Suppl 1:S7.

Kalisch M, B"{u}hlmann P. Estimating High-Dimensional Directed Acyclic Graphs with the PC-Algorithm. J Mach Learn Res. 2007;8.

Markowetz F, Bloch J, Spang R. Non-transcriptional pathway features reconstructed from secondary effects of RNA interference. Bioinformatics. 2005;21(21):4026–4032.

Pirkl M, Hand E, Kube D, Spang R. Analyzing synergistic and non-synergistic interactions in signalling pathways using Boolean Nested Effect Models. Bioinformatics. 2016;32(6):893–900.

van Wageningen S, Kemmeren P, Lijnzaad P, Margaritis T, Benschop JJ, de Castro IJ, et al. Functional overlap and regulatory links shape genetic interactions between signaling pathways. Cell. 2010 Dec;143(6):991–1004.

Sameith K, Amini S, Groot-Koerkamp MJA, van Leenen D, Brok M, Brabers N, et al. A high-resolution gene expression atlas of epistasis between gene-specific transcription factors exposes potential mechanisms for genetic interactions. BMC biology. 2015 Dec;13:112.

Guangchuang Yu, Fei Li, Yide Qin, Xiaochen Bo, Yibo Wu, Shengqi Wang; GOSemSim: an R package for measuring semantic similarity among GO terms and gene products. Bioinformatics 2010; 26 (7): 976-978. doi: 10.1093/bioinformatics/btq064
